# Supplementary material for: Annotating pathogenic non-coding variants in genic regions
Source: Nat Commun. 2017 Aug 9;8:236. doi: 10.1038/s41467-017-00141-2 (PMC5550444; doi:10.1038/s41467-017-00141-2)
Supplement: Supplementary file 1 — Supplementary Information [file 41467_2017_141_MOESM1_ESM.pdf]

File name: Supplementary Information
Description: Supplementary figures, supplementary notes, supplementary methods and supplementary references.

File name: Supplementary data 1
Description: Model Features and Additional Variant Information calculated by TraP.

File name: Supplementary data 2
Description: Training-set synonymous variants.

File name: Supplementary data 3
Description: Feature importance.

File name: Supplementary data 4
Description: OCD DNMs.

File name: Supplementary data 5
Description: Sizes of test datasets.

File name: Supplementary data 6
Description: Epi4K DNMs.

File name: Peer review file
Description:

Supplementary Note 1. TraP Score Features' Importance.

The final TraP model is using 20 selected features (Supplementary Data 1 and Methods) and the importance measurements of each feature's contribution were measured (Supplementary Data 3). While there is no specific feature that is exceptionally high, we find that features that strongly contribute to the final TraP pathogenicity predictions are mostly designed to capture interactions between various variant attributes. The strongest contributor is the Variant Splice Score (F20, Supplementary Data 1) that incorporates original and cryptic splice site changes as well as regulatory changes and conservation. The 2nd contributing feature is the GERP++ conservation score, followed by the Variant Regulatory Score (F19, Supplementary Data 1), that incorporates mostly regulatory features. Also important are the Splice Site Overall Score (F11, Supplementary Data 1) that incorporates 3' and 5' splice site negative effects and the Cryptic 5' Splice Site Score (F7, Supplementary Data 1), an independent feature corresponding to the strength of a newly created 5' splice site and points to the stronger contribution of variants that create new 5' versus 3' splice sites.

Supplementary Note 2. TraP Variants Are Not Predominantly Located Near Training-Set Variants.

We examined whether the 2.16% TraP-predicted pathogenic variants are located predominantly near the training set pathogenic variants. To answer this question, we first considered the local sequence content flanking pathogenic-assigned training-set variants. Within 100 flanking bases and subsequently within 1,000 and 10,000 flanking bases the percentage of TraP pathogenic-predicted variants are 0.012% ± 0.033, 0.035% ± 0.057 and 0.15% ± 0.23, respectively. We then did similar for 1,000 randomly selected TraP-predicted pathogenic synonymous variants and found that the neighboring 100 flanking bases and subsequently 1,000 and 10,000 bases had comparable rates of TraP-predicted pathogenicity of 0.012% ± 0.034, 0.035% ± 0.069 and 0.13% ± 0.19, respectively. All three distance groups (100bp, 1,000bp and 10,000bp) are not statistically different between the training set and the randomly selected TraP-predicted pathogenic variants. Thus, training-set variants have similar local rates of TraP pathogenicity scores as pathogenic-predicted variants.

Supplementary Note 3. DNMs Have Similar TraP Scores as General Population.

Since TraP is trained on *de novo* mutations (DNMs), we needed to assure that TraP does not select against specific properties of DNMs. For this purpose, we analyzed exomes from 436 Obsessive Compulsive Disorder (OCD) family trios. Using very strict criteria we identified 97 synonymous DNMs that were scored using TraP (Supplementary Data 4, see Methods). OCD DNMs have an average TraP of 0.093, significantly higher than the training-set control DNMs (MW-test, p-value = 1.2×10^{-08}), yet similar to the TraP average of 1.46M ExAC synonymous variants (MW-test, p-value = 0.69). This assures that TraP does not select against features specifically related to DNMs.

Supplementary Note 4. Other Tools for Assessing Variants' Effects on the Transcript.

Other methods, such as the SPANR[1] and SilVA[2] tools provide valuable predictions for transcript changes originating from splicing. SPANR is focused on predicting the inclusion level of a middle exon in an exon-triplet and can predict cassette-exon skipping events with very high accuracy. That said, many types of substitutions will disrupt a fraction of the transcripts, causing either a loss or even a gain of function that will not be accounted for in the inclusion level of a middle exon (intron retention/alternative junctions), or it will be discounted as a minor change in inclusion level. TraP is not aiming to predict the changes to the final transcript but the fact that a change will occur. The SilVA tool is more similar to TraP in design, yet differs in feature calculations and choice of training sets and focuses solely on synonymous variants. While having high specificity, it's reported sensitivity is lower than measured by minimum TraP threshold on synonymous data, yet very much acceptable for scoring synonymous variants for pathogenic effects.

Supplementary Methods

OCD dataset

The OCD dataset consists of 436 OCD family trios. 97 *de novo* synonymous variants were extracted based on the same criteria as mentioned above for Epi4K DNMs (Supplementary Data 4).

Features calculation

Below are the calculations used to compute all 20 features that were used in the TraP model:

F1: Variant Status Factor. 1 – the variant position resides only within exons or only within introns in all the genes' transcripts, 2 – the variant position resides in exons in some transcripts and in introns in other transcripts.

F2: Number of Affected Transcripts. The number of transcripts of the gene harboring the variant that can be affected by it. If a gene's transcript does not harbor the position of the variant, it is not included in the count.

F3: Within 5'ss Region. 0 - the variant is within the 5'ss region, i.e: last 3 exonic bp and first 6 downstream intronic bp. 1 – the variant is outside the 5'ss region.

F4: Within 3'ss Region. 0 - the variant is within the 3'ss region, i.e: last 20 upstream intronic bp and first 3 exonic bp. 1 – the variant is outside the 3'ss region.

F5: Number of ESR and SS Enhancer Cases. The count of events that might positively affect inclusion: variants that cause a creation of binding sites for enhancer Splicing Regulatory Proteins (SRPs), variants that cause a disruption of binding sites to silencer SRPs and variants that strengthen an existing splice site.

F6: Cryptic 3'ss score w/ variant. If the variant creates a new 'AG' dinucleotide, the PSSM score around this new dinucleotide is computed as explained in the Methods section.

F7: Cryptic 5'ss score w/ variant. If the variant creates a new 'GT' dinucleotide, the PSSM score around this new dinucleotide is computed as explained in the Methods section.

F8: GERP++ RS. The GERP++ RS conservation score as obtained from the hg19 GERP database for the position of the variant in question.

The twelve features calculated below are designed to capture underlying interactions between non-independent attributes that affect the transcript synergistically rather than as single features. An example to this would be a variant that is creating a new strong cryptic splice site, the effect of such a variant might be different depending on the score of the exons' original splice site. The original splice site is considered as a variant attribute, and therefore this interaction between new and original splice site is an interaction between the variant's attributes. Using only the score of the splice site (both new and original) proved too weak when constructing the model, yet the interactions strengthened the model significantly. The following section explains the equations and rationale behind these more complex features:

F9 and ***F10***: 3'ss and 5'ss Silencer Scores. The score is a sum of difference across all transcripts between the reference computed splice site PSSM score and the alternative splice site PSSM score in case the following conditions apply: 1) the variant is within the splice site region and 2) the new splice site is stronger than the original splice site. The rational here is that the effect will correlate with the difference between splice sites and not their absolute strength.

$$\mathrm{F9} = \sum_{i=1}^{tmax} (Alt3_i - Ref3_i)$$

$$\mathrm{F10} = \sum_{i=1}^{tmax} (Alt5_i - Ref5_i)$$

Where *tmax* is the number of transcripts for a specific gene, *Ref3 and Ref5* are the PSSM scores for the reference 3' and 5' splice sites in the i-th transcript respectively, *Alt3* and *Alt5* are the PSSM scores for the alternative 3' and 5' splice sites in the i-th transcript. F_9 and F_10 are the silencing scores for each splice site.

F11: Splice Site Overall score. The sum of features F9 and F10. The total effect of the variant on both 3' and 5' splice sites across all transcripts of the harboring gene.

F12: Silencing Effect Score: Overall difference between reference splice site PSSM score and new splice site score combining the information from both 3' and 5' splice sites across all transcripts. Here we calculate the interaction between the overall difference of new stronger splice site and the original splice site across all the transcripts of the gene, while taking into account normalized PSSM scores. The rationale behind this formula is that a variant will affect all transcripts of a gene and will be dependent in the relative strength of the strongest splice site.

$$min3 = 22.995$$
$$min5 = 32.083$$

$$norm3 = \frac{\max(Ref3, Alt3)}{min3}$$

$$norm5 = \frac{\max(Ref5, Alt5)}{min5}$$

$$\text{F12} = \sum_{i=1}^{tmax} (norm3 \times (Alt3_i - Ref3_i) + norm5 \times (Alt5_i - Ref5_i))$$

$$TES = \sum_{i=1}^{tmax} (norm3 \times \text{abs}(Ref3_i - Alt3_i) + norm5 \times \text{abs}(Ref5_i - Alt5_i))$$

Where *tmax* is the number of transcripts for the specific gene, *min3* and *min5* are the PSSM scores for the 3' and 5' splice sites without a canonical splice site (used to weight the scores according to fold change from minimum score), *Ref3 and Ref5* are the PSSM scores for the reference 3' and 5' splice sites, *Alt3* and *Alt5* are the PSSM scores for the alternative 3' and 5' splice sites, *F12* is the silencing effect score and TES is a total effect score used later in the construction of the Variant Splice Score (F20).

F13 and ***F14***: Cryptic 3'ss and 5'ss Effect Scores: these features calculate a score for a new cryptic splice site. The score is made of the PSSM score difference between a newly created/disrupted 3'/5' splice site and the original sequence at that position.

$$F13 = c3alt - c3ref$$

$$F14 = c5alt - c5ref$$

Where *c3Alt* and *c5Alt* are the cryptic PSSM scores calculated for the variant, *c3Ref* and *c5Ref* are the PSSM scores calculated for the reference sequence, F13 and F14 are the final effect scores.

F15 and ***F16***: Cryptic 3'ss and 5'ss Enhancer Scores. Similar to 3'ss and 5'ss Cryptic Effects (F13-F14), but with only positive differences examined, thus setting a zero value to features of variants that create cryptic splice sites that are weaker than the reference.

F17 and ***F18***: Splicing regulatory binding site scores. TraP uses four datasets of major splicing regulatory proteins: SRSF1[3], SRSF2[4], SRSF5[3] and SRSF6[3], and one set of splicing silencer sequences calculated in-silico [5]. For each of these proteins, TraP is using a normalized rank score (between 0 and 1) indicating the ranking of a given sequence relative to the other sequences identified for that protein. The ranked datasets were obtained from Schwartz et al. [6] and correspond to the sequences' p-values or PSSM log-odd scores. For each variant, TraP identifies creations or disruptions of sequences and uses the rank of the sequence to calculate the related feature. Thus, a creation of an enhancer sequence that ranks 0.9 will add +0.9 to an ESR enhancer score, while a creation of a silencer sequence that ranks 0.5 will add +0.5 to an ESR silencer score. Eventually, the ESR enhancer score will be the addition of all the creations of enhancers and disruptions of silencers for the five datasets. The ESR silencer score, accordingly, will be creations of silencers and disruptions of enhancers.

$$eeS = \left(\sum_{i=1}^{EGroups} \prod_{j=1}^{ESRs} VR_{ij} \right) + \left(\sum_{i=1}^{SGroups} \prod_{j=1}^{ESRs} RR_{ij} \right)$$

$$esS = \left(\sum_{i=1}^{EGroups} \prod_{j=1}^{ESRs} RR_{ij} \right) + \left(\sum_{i=1}^{SGroups} \prod_{j=1}^{ESRs} VR_{ij} \right)$$

$$F17 = esS - eeS$$

$$F18 = esS + eeS$$

Where EGroups are groups of Enhancer SRPs, ESRs are the sequences in each SRP datasets, RR is the rank of a reference sequence that was disrupted, VR is the rank of a sequence created by the variant, *eeS* is the ESR enhancer score and e*sS* is the ESR silencer score. F17 is the negated ESR score, that is the overall tendency of the variant to either silencing or enhancing, calculated as subtraction of the enhancing events caused by the variant from the silencing events caused by it. F18 is the combined ESR score that holds the overall disturbance of regulatory sequences caused by the variant, calculated as the addition of all enhancing and silencing ESR events caused by the variant.

F19: Variant Regulatory Score. This score combines all the effects caused by the variant that do not directly change the splice site region. Therefore, the following equations are using variant attributes calculated previously in other features.

$$minCanonical3 = 44.836$$
$$minCanonical5 = 62.556$$

$$cryp = \left(F13\frac{\mathrm{c3ss}}{100 - minCanonical3}\right) + \left(F14\frac{\mathrm{c5ss}}{100 - minCanonical5}\right)$$

$$esr = esS * sCount + eeS * eCount$$

$$F19 = esr \times F1 + F8 + cryp$$

Where *c3ss* and *c5ss* are cryptic splice sites scores (features F6 and F7), c3ES and c5ES are the cryptic effect scores (features F13 and F14), *cryp* is the calculated effect of cryptic splice site creation, esS and eeS are ESR enhancer and silencer scores (calculated for features F17 and F18), *sCount* is the number of silencing events, *eCount* is the number of enhancing events, *esr* is the total effect of ESR attributes, *F1* is the Variant Status Factor, *F8* is the GERP RS conservation score and VRS is the final Variant Regulatory Score.

F20: Variant Splice Score: the combined effects and interactions of regulatory and splice region features. The subtraction of the Variant Status Factor is acting to reduce the effect of a variant that resides in an alternatively spliced exon.

$$splice = F9 + F10 + \ F12 + TES - F1$$

$$F20 = esr + F8 + \ cryp \ + \left(\frac{\mathrm{F13}}{minCanonical3}\right) + \left(\frac{\mathrm{F14}}{minCanonical5}\right) + splice$$

Where *F9* and *F10* are the silencer scores, F12 and TES are the silencing and total effect scores, *splice* is the total addition of splice region effects, *esr* is the total effect of ESR attributes (calculated for F19), *F8* is the GERP RS conservation score, *cryp* is the effect of cryptic splice site creation (calculated for F19), *F13* and *F14* are the cryptic effect scores and F20 is the final Variant Splice Score that incorporates attributes for splice junction, ESRs, conservation and cryptic splice sites.

Alternative TraP Model

An alternative TraP model was constructed using the training set 75 curated pathogenic variants and additional 66 un-curated synonymous variants obtained from ClinVar (used as a test set for figure 3A). A total of 141 pathogenic-assigned training variants was used for the alternative model. The alternative model with the un-curated variants had a lower specificity than the model with only the curated 75 pathogenic variants, miss-classifying 9/402 benign training variants as pathogenic, compared to only 4/402 with the original model. Since TraP was constructed with an aim for highest specificity, this alternative model was not further evaluated.

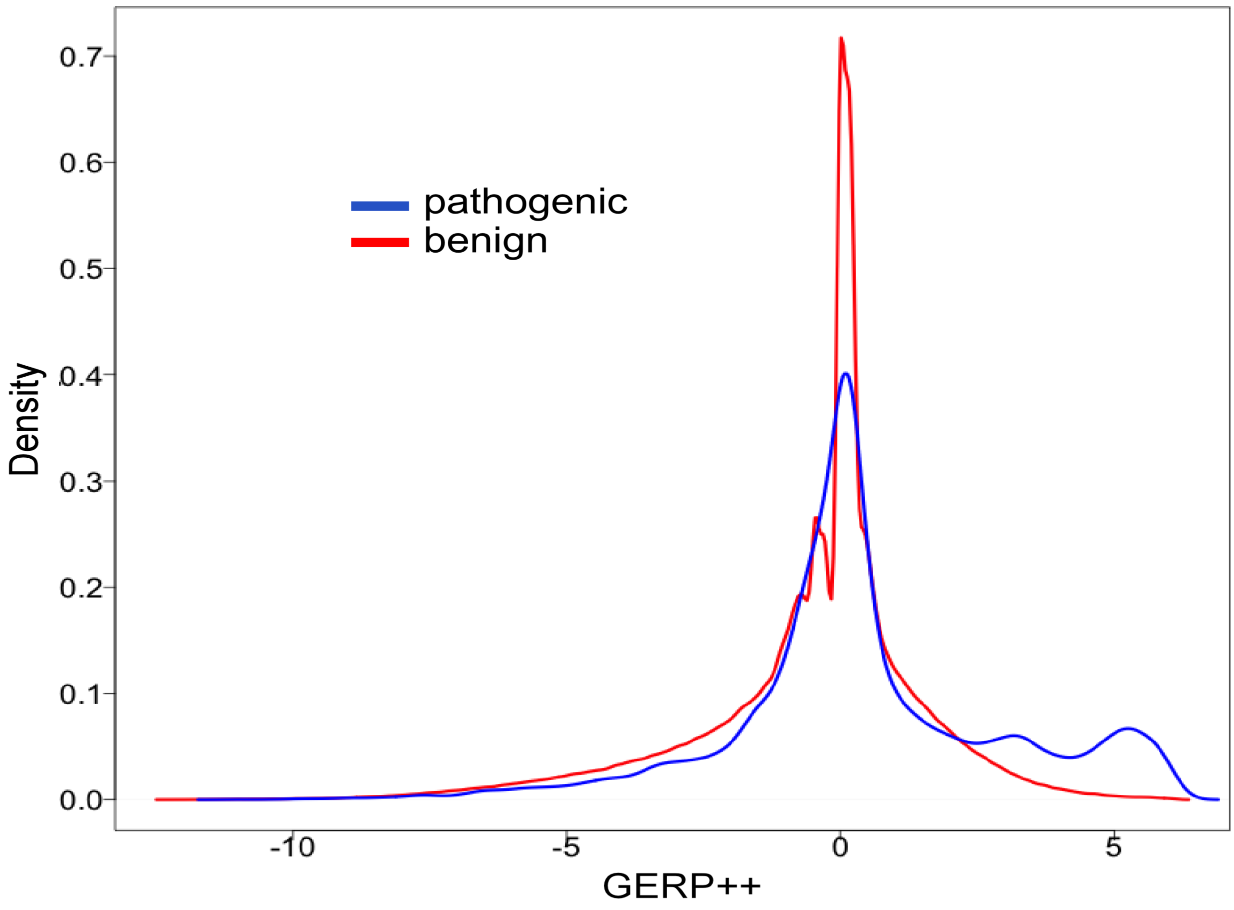

Supplementary Figure 1. GERP++ distribution of TraP considered pathogenic and benign intronic variants. GERP++ score for these 1.5M random intronic variants that are considered pathogenic (blue) or benign (red) by TraP. Median GERP++ score for intronic variants considered pathogenic by TraP (TraP ≥ 0.459) is 0.12, suggesting that half of them are not under evolutionary constraints.

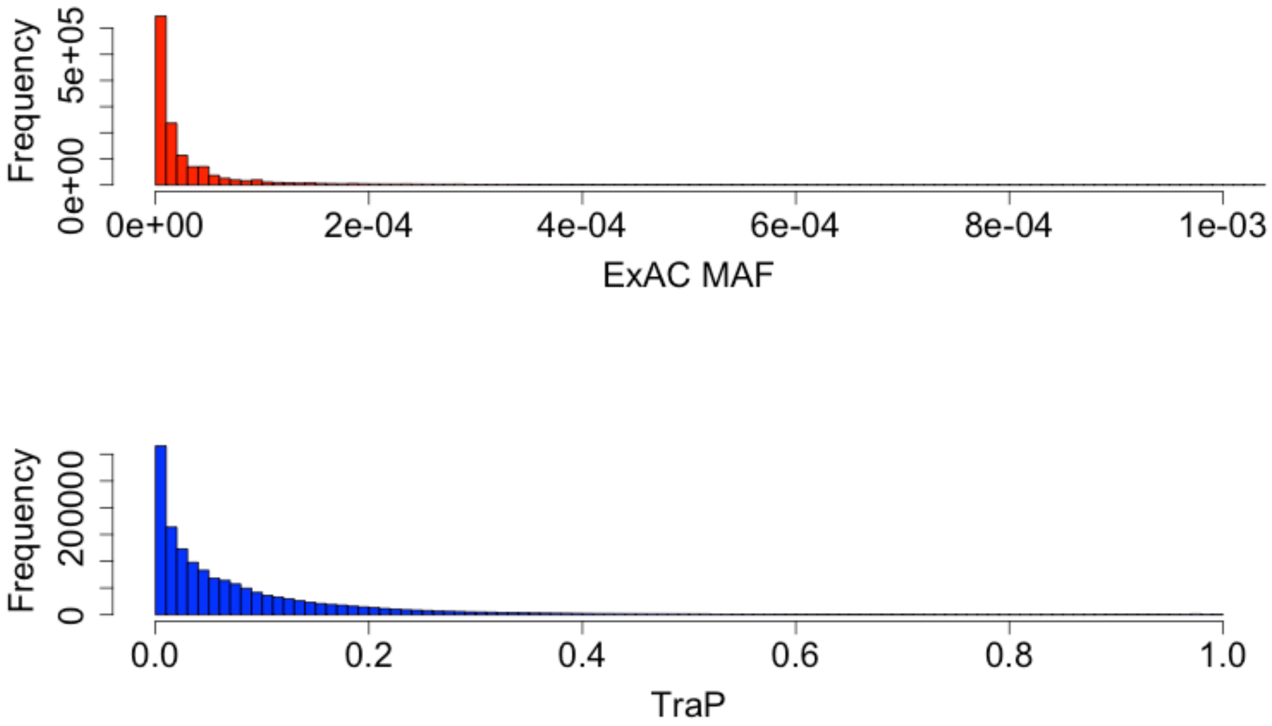

Supplementary Figure 2. ExAC MAF and TraP distributions for ExAC 1.46M synonymous variants. Both ExAC MAF (red) and TraP (blue) distributions are highly positively skewed with most values clustered around zero.

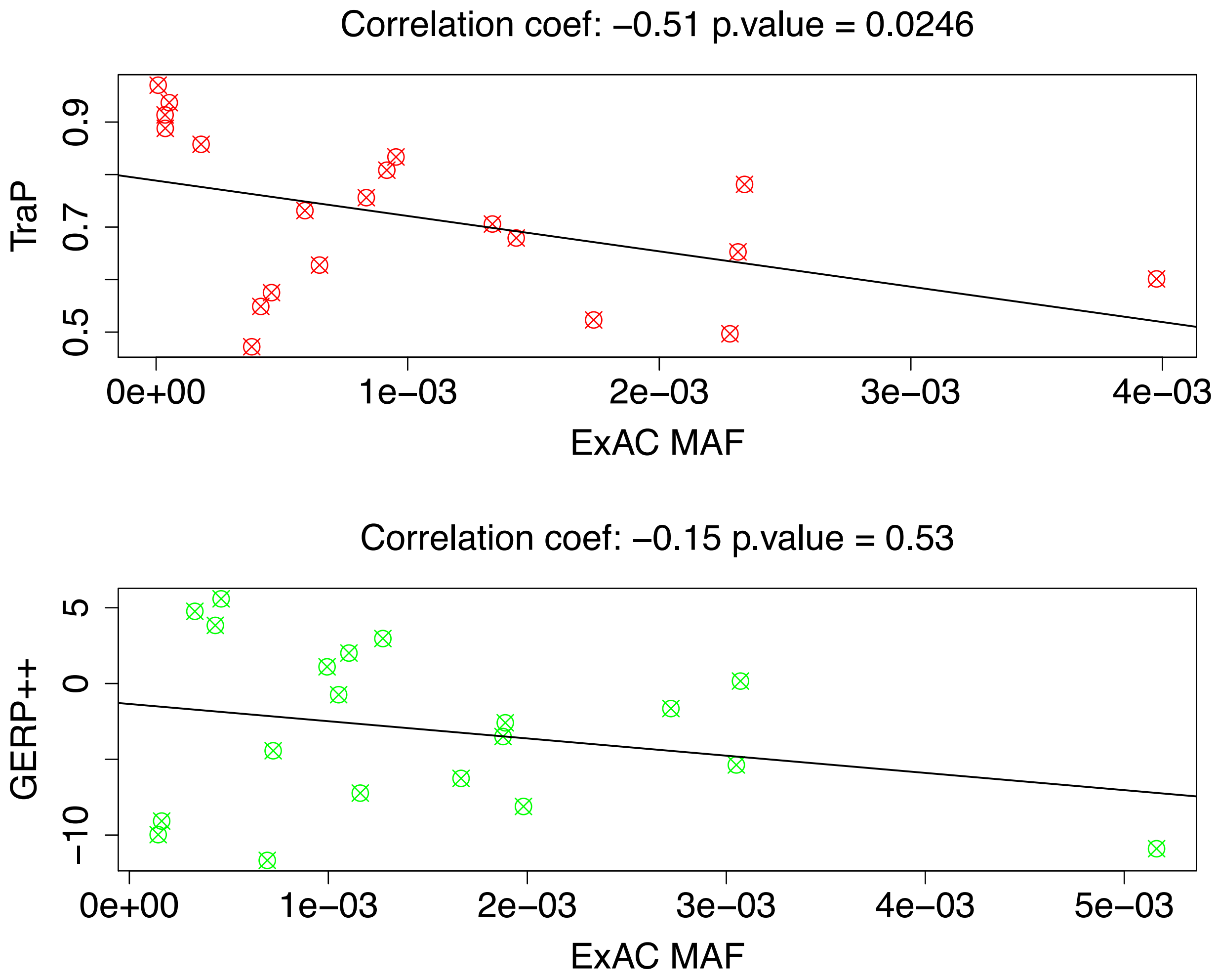

Supplementary Figure 3. Correlation of TraP and GERP++ scores with allele frequency. Correlation between TraP score and MAF for cryptic splice site variants that are predicted as pathogenic by TraP (upper panel, TraP ≥ 0.459, 6,328 variants). The dataset was binned into 20 groups by taking 5% score intervals and examining the correlation of the 20 points with the average MAF for each group. TraP correlation is −0.51 (p-value = 0.025) while GERP++ correlation of the same dataset is −0.15 (lower panel, green points, p-value = 0.53). TraP can help identify potential variants that are deleterious to the transcript, but do not necessarily have a strong conservation signature.

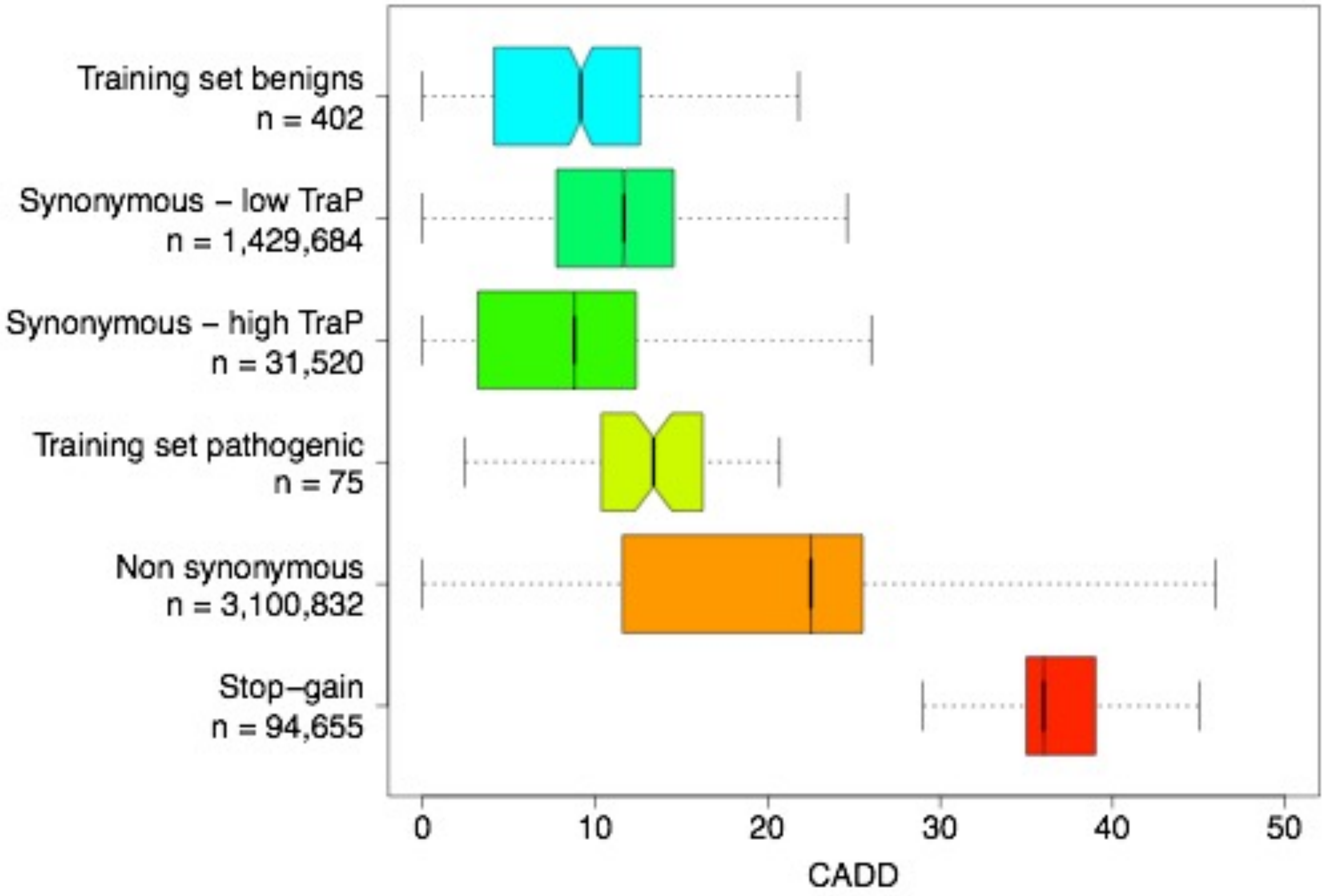

Supplementary Figure 4. CADD score for various variant types. CADD considers the known pathogenic variants from the training set (yellow, CADD 12.88 ± 4.95) as significantly less damaging than NS variants (orange, CADD 18.67 ± 9.84). The whiskers of the boxplots extend to the most extreme data point, which is no more than 1.5 times the interquartile range away from the box.

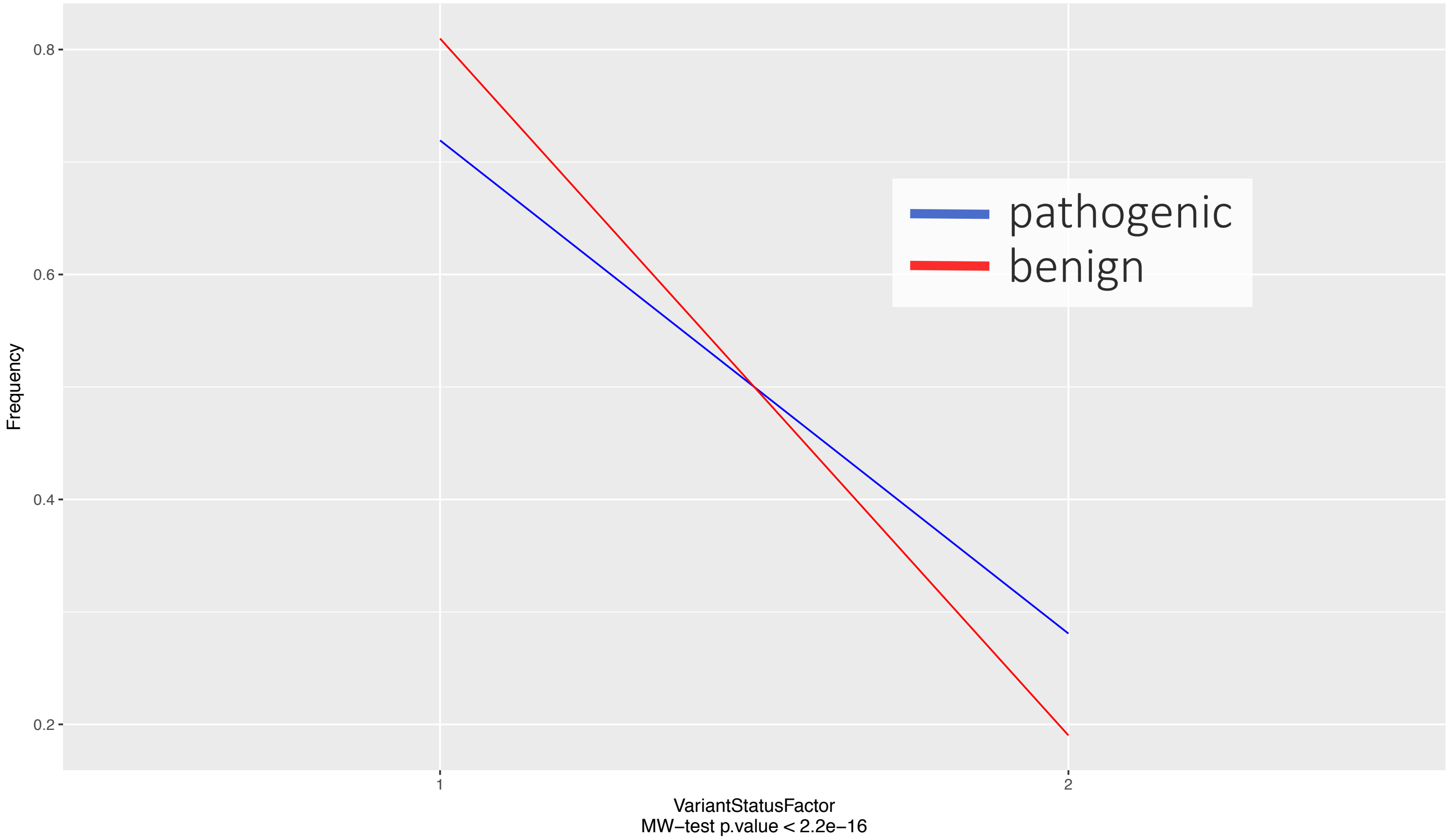

Supplementary Figure 5. Frequency distribution of the Variant Status Factor (feature F1) for ExAC 1.46M synonymous variants. Frequencies are presented for TraP-predicted pathogenic variants (blue, TraP ≥ 0.459) and TraP-predicted benign variants (red, TraP < 0.459). X-axis represents the Variant Status Factor: 1 – the variant position resides only within exons or only within introns in all the genes' transcripts, 2 – the variant position resides in exons in some transcripts and in introns in other transcripts. Corresponding Mann Whitney U test is used to compare benign and pathogenic variant distributions (bottom line).

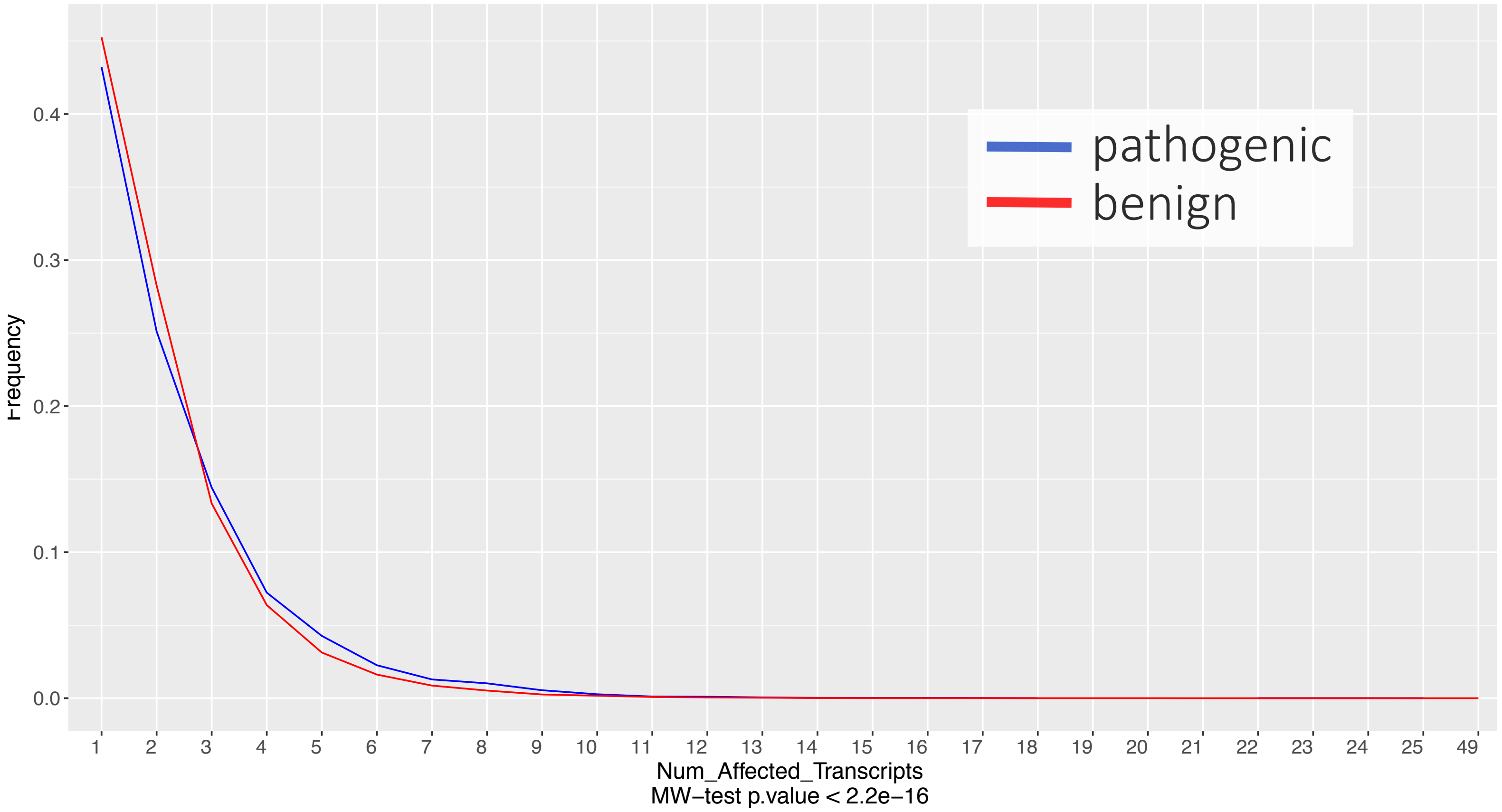

Supplementary Figure 6. Frequency distribution of the Number of Affected Transcripts (feature F2) for ExAC 1.46M synonymous variants. Frequencies are presented for TraP-predicted pathogenic variants (blue, TraP ≥ 0.459) and TraP-predicted benign variants (red, TraP < 0.459). X-axis represents the Number of Affected Transcripts: the number of transcripts of the gene harboring the variant that can be affected by it. If a gene's transcript does not harbor the position of the variant, it is not included in the count. Corresponding Mann Whitney U test is used to compare benign and pathogenic variant distributions (bottom line).

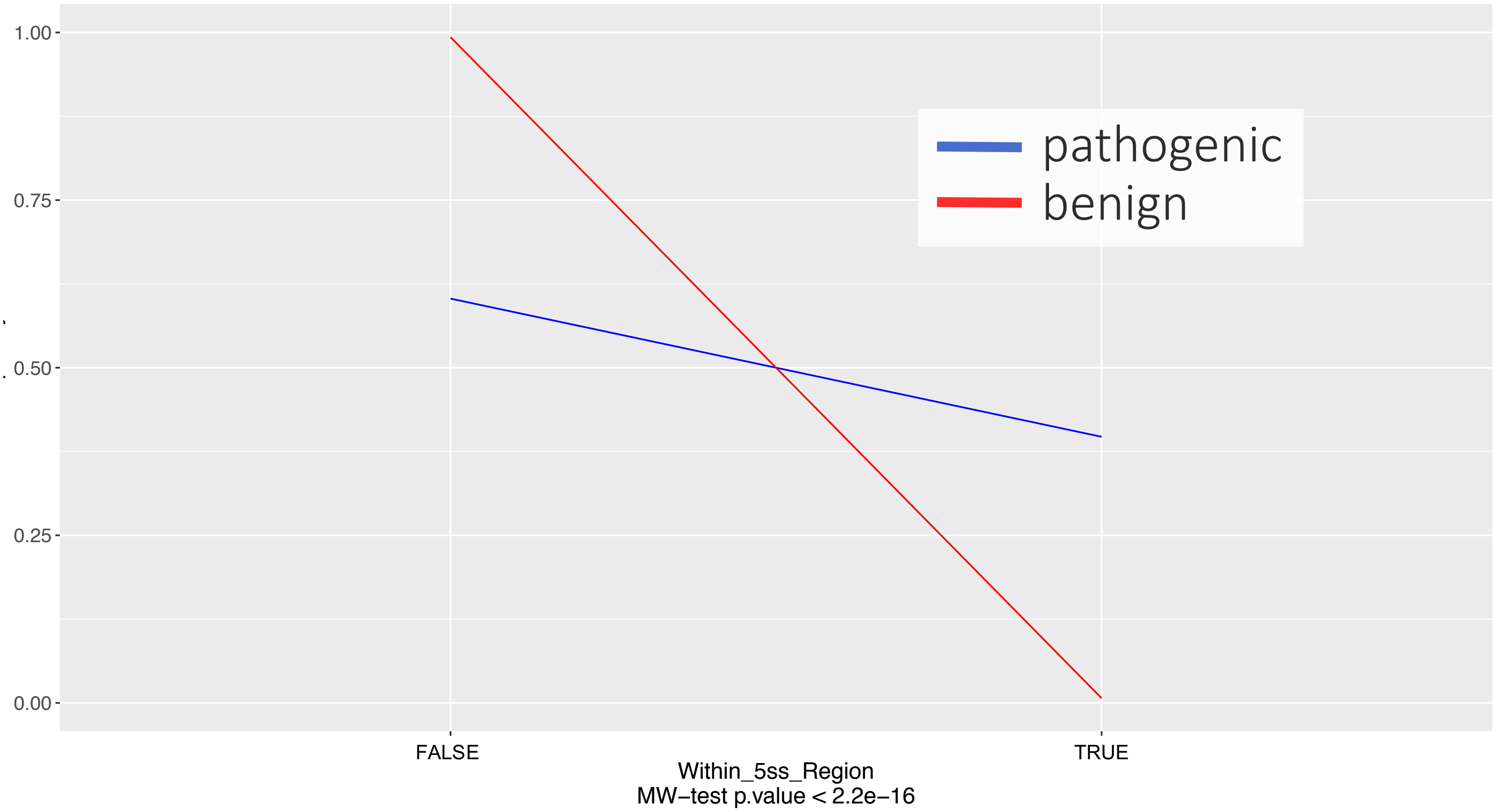

Supplementary Figure 7. Frequency distribution of the Within 5’ss Region feature (feature F3) for ExAC 1.46M synonymous variants. Frequencies are presented for TraP-predicted pathogenic variants (blue, TraP ≥ 0.459) and TraP-predicted benign variants (red, TraP < 0.459). X-axis represents the values of the Within 5’ss Region feature: 0 - the variant is within the 5’ss region, i.e: last 3 exonic bp and first 6 intronic bp. 1 – the variant is outside the 5’ss region. Corresponding Mann Whitney U test is used to compare benign and pathogenic variant distributions (bottom line).

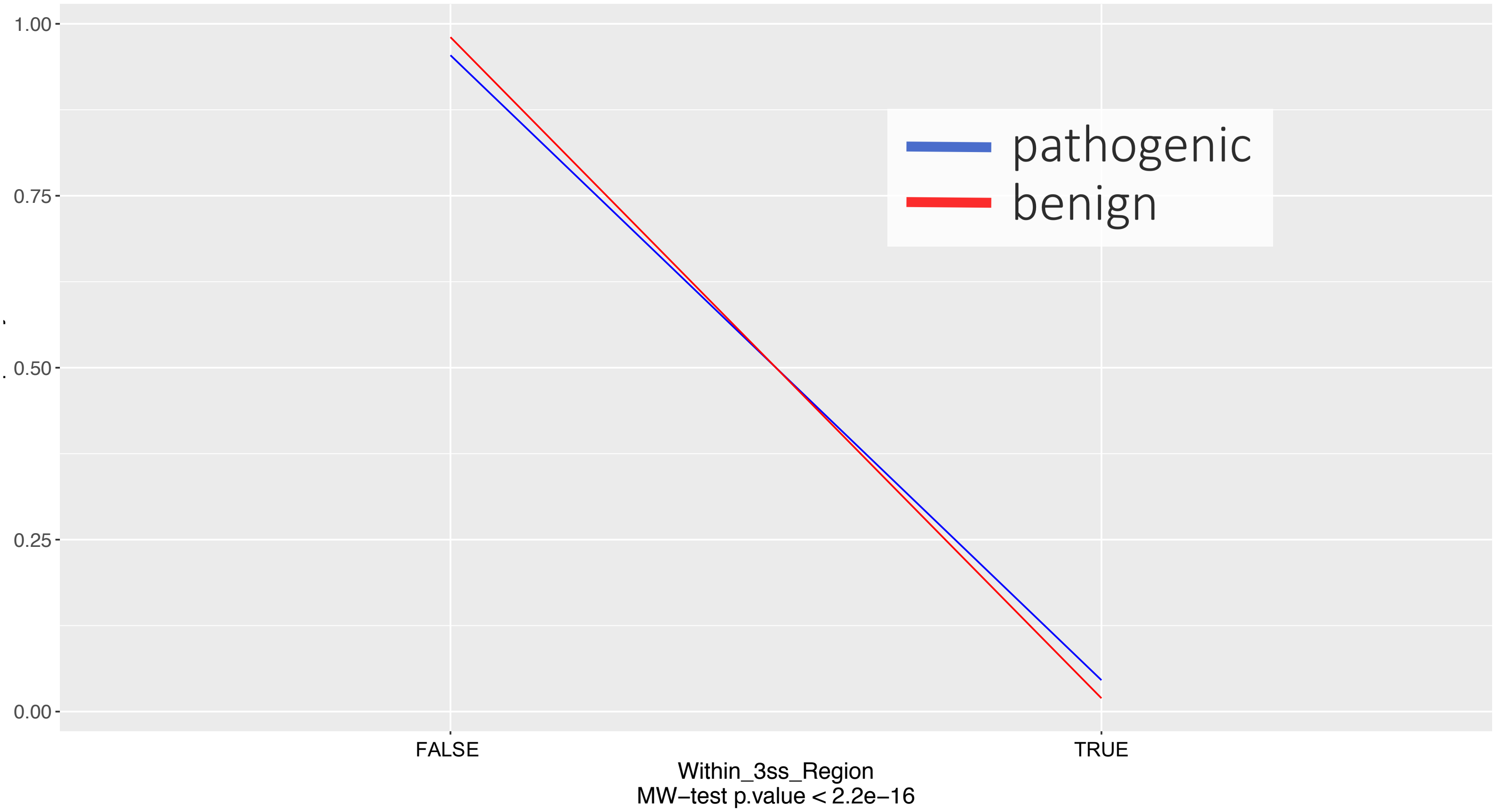

Supplementary Figure 8. Frequency distribution of the Within 3’ss Region feature (feature F4) for ExAC 1.46M synonymous variants. Frequencies are presented for TraP-predicted pathogenic variants (blue, TraP ≥ 0.459) and TraP-predicted benign variants (red, TraP < 0.459). X-axis represents the values of the Within 3’ss Region feature: 0 - the variant is within the 3’ss region, i.e: last 20 upstream intronic bp and first 3 exonic bp. 1 – the variant is outside the 3’ss region. Corresponding Mann Whitney U test is used to compare benign and pathogenic variant distributions (bottom line).

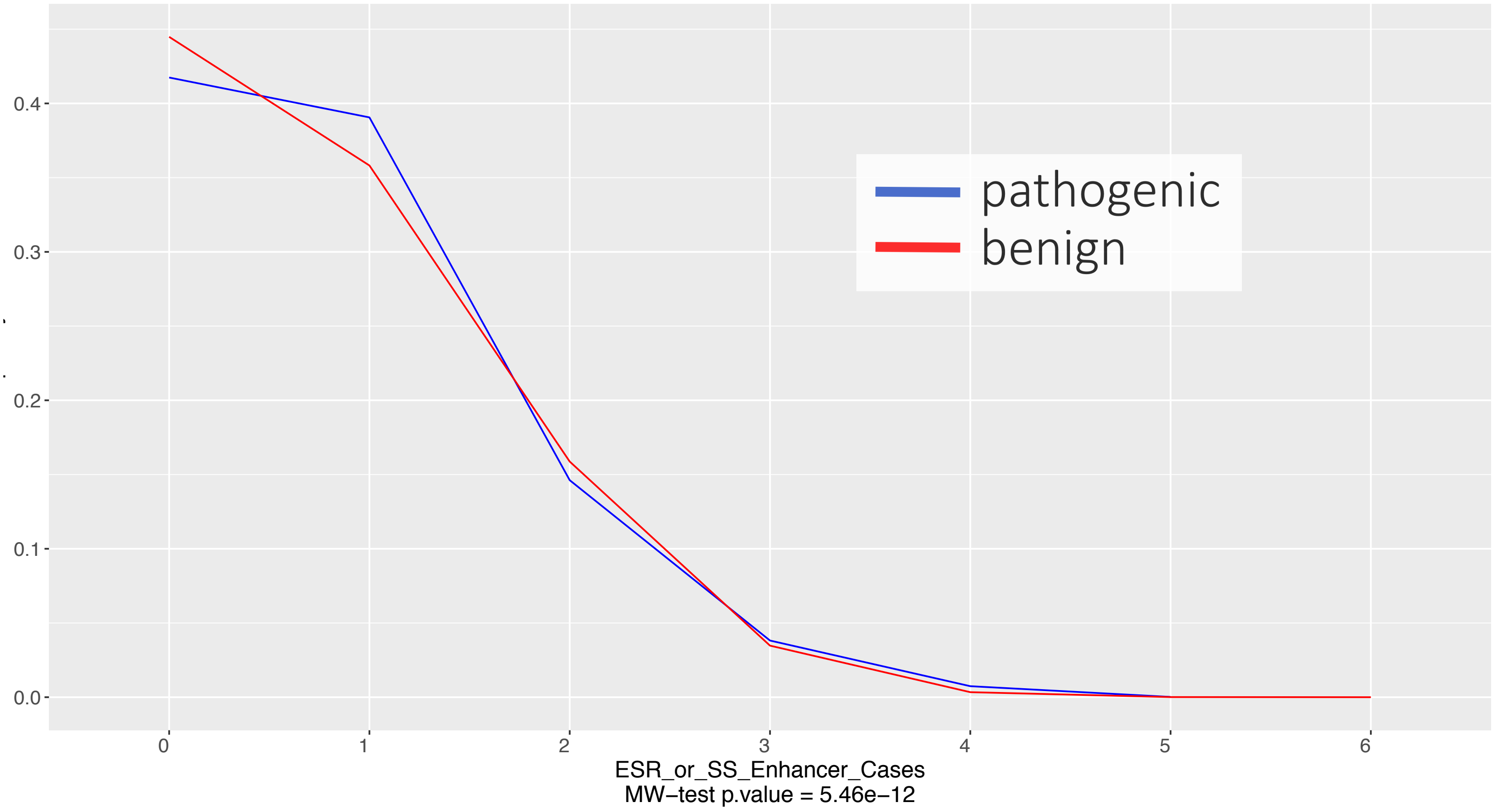

Supplementary Figure 9. Frequency distribution of the Number of ESR and SS Enhancer Cases (feature F5) for ExAC 1.46M synonymous variants. Frequencies are presented for TraP-predicted pathogenic variants (blue, TraP ≥ 0.459) and TraP-predicted benign variants (red, TraP < 0.459). X-axis represents the Number of ESR and SS Enhancer Cases: the count of events that might positively affect inclusion: variants that cause a creation of binding sites for enhancer SRPs, variants that cause a disruption of binding sites to silencer SRPs and variants that strengthen an existing splice site. Corresponding Mann Whitney U test is used to compare benign and pathogenic variant distributions (bottom line).

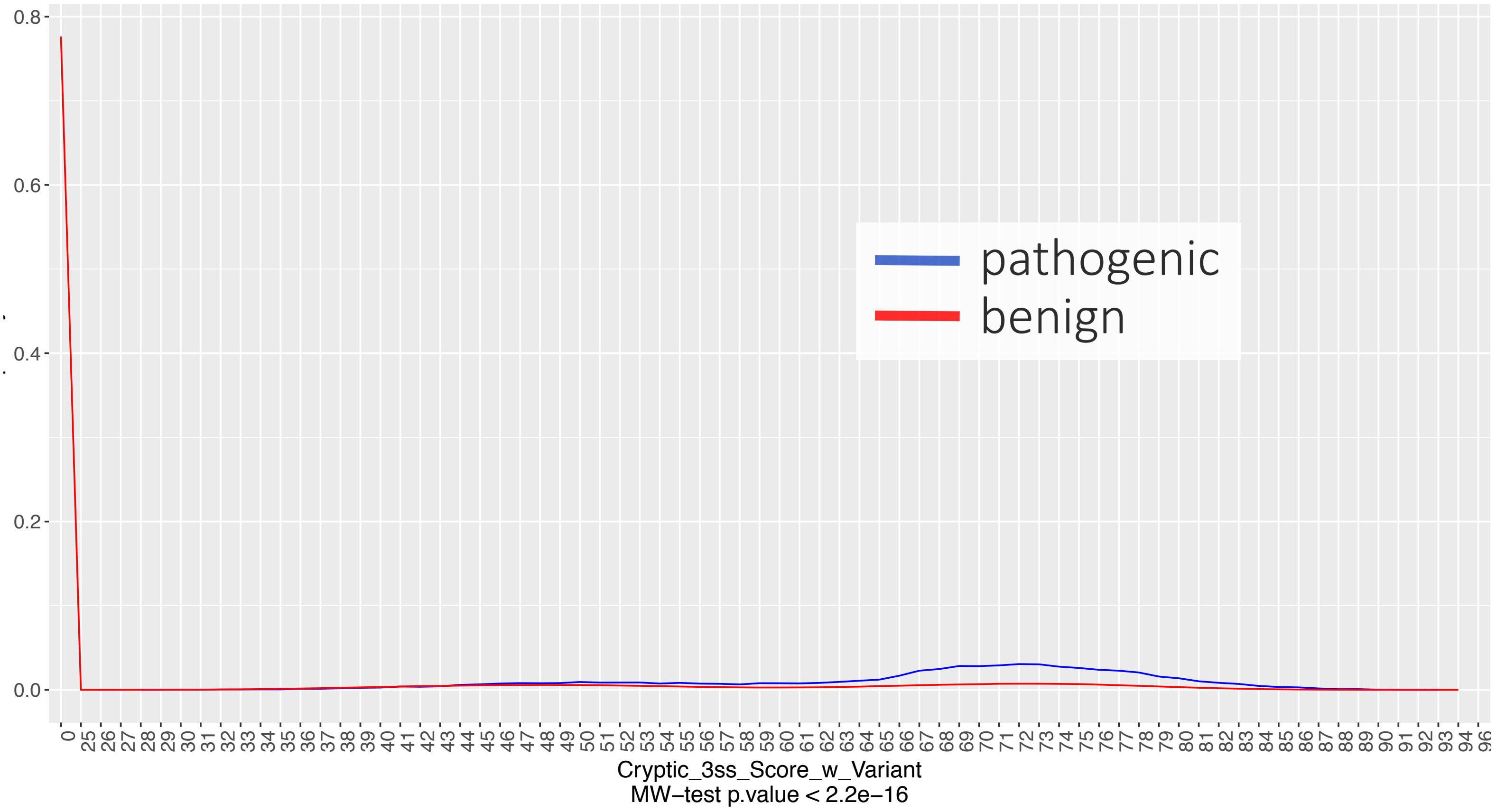

Supplementary Figure 10. Frequency distribution of the Cryptic 3'ss score w/ variant (feature F6) for ExAC 1.46M synonymous variants. Frequencies are presented for TraP-predicted pathogenic variants (blue, TraP ≥ 0.459) and TraP-predicted benign variants (red, TraP < 0.459). If the variant creates a new 'AG' dinucleotide, the PSSM score around this new dinucleotide is computed. The X-axis represents the score of this new cryptic splice site. Corresponding Mann Whitney U test is used to compare benign and pathogenic variant distributions (bottom line).

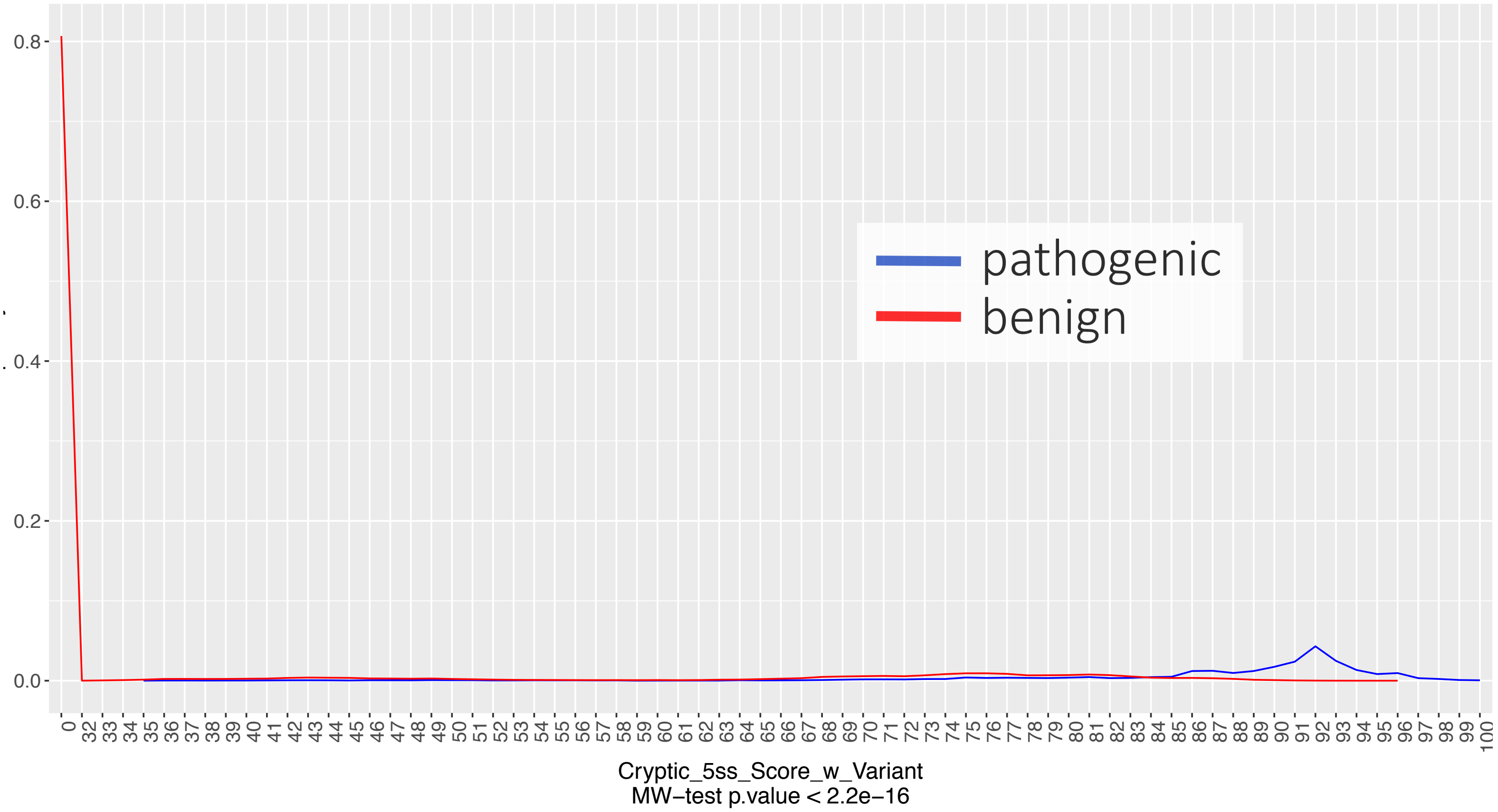

Supplementary Figure 11. Frequency distribution of the Cryptic 5'ss score w/ variant (feature F7) for ExAC 1.46M synonymous variants. Frequencies are presented for TraP-predicted pathogenic variants (blue, TraP ≥ 0.459) and TraP-predicted benign variants (red, TraP < 0.459). If the variant creates a new 'GT' dinucleotide, the PSSM score around this new dinucleotide is computed. The X-axis represents the score of this new cryptic splice site. Corresponding Mann Whitney U test is used to compare benign and pathogenic variant distributions (bottom line).

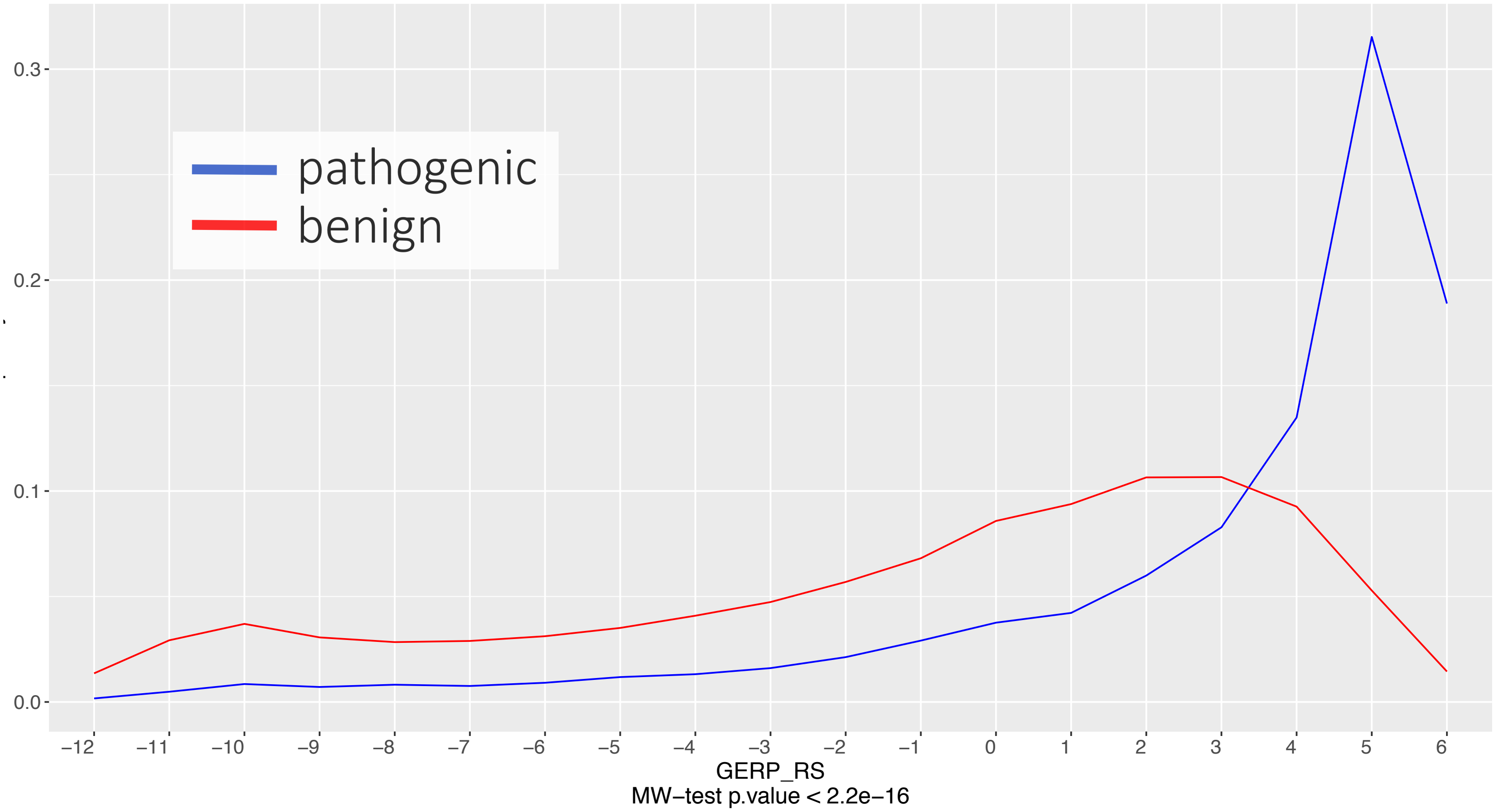

Supplementary Figure 12. Frequency distribution of the GERP++ RS score (feature F8) for ExAC 1.46M synonymous variants. Frequencies are presented for TraP-predicted pathogenic variants (blue, TraP ≥ 0.459) and TraP-predicted benign variants (red, TraP < 0.459). The X-axis represents the GERP++ RS conservation score as obtained from the hg19 GERP database for the position of the variant in question. Corresponding Mann Whitney U test is used to compare benign and pathogenic variant distributions (bottom line).

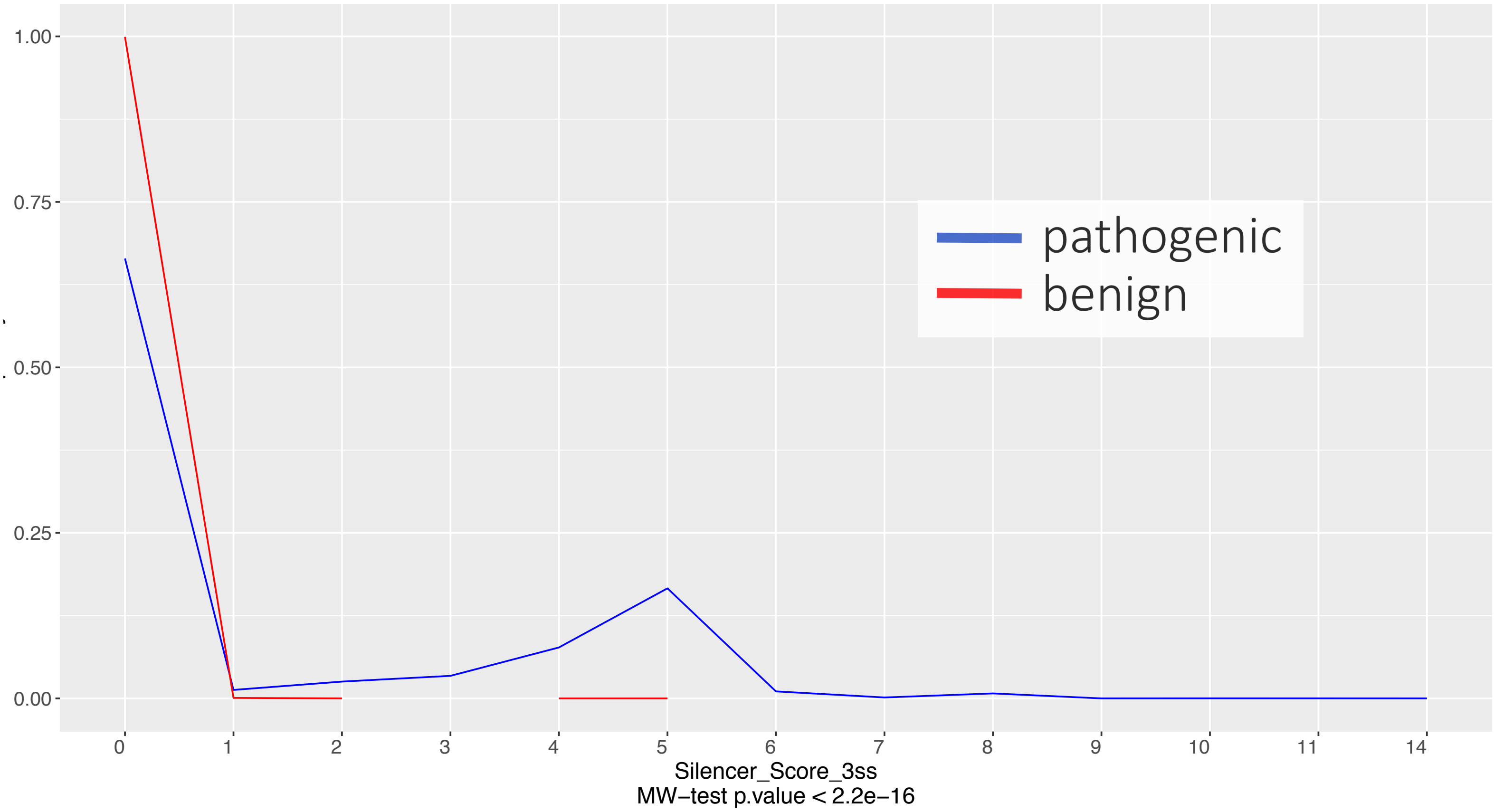

Supplementary Figure 13. Frequency distribution of the 3’ss Silencer Score (feature F9) for ExAC 1.46M synonymous variants. Frequencies are presented for TraP-predicted pathogenic variants (blue, TraP ≥ 0.459) and TraP-predicted benign variants (red, TraP < 0.459). The X-axis represents the 3’ss Silencer Score. The score is a sum of difference across all transcripts between the reference computed 3’ splice site PSSM score and the alternative 3’ splice site PSSM score in case the following conditions apply: 1) the variant is within the splice site region and 2) the new splice site is stronger than the original splice site. Corresponding Mann Whitney U test is used to compare benign and pathogenic variant distributions (bottom line).

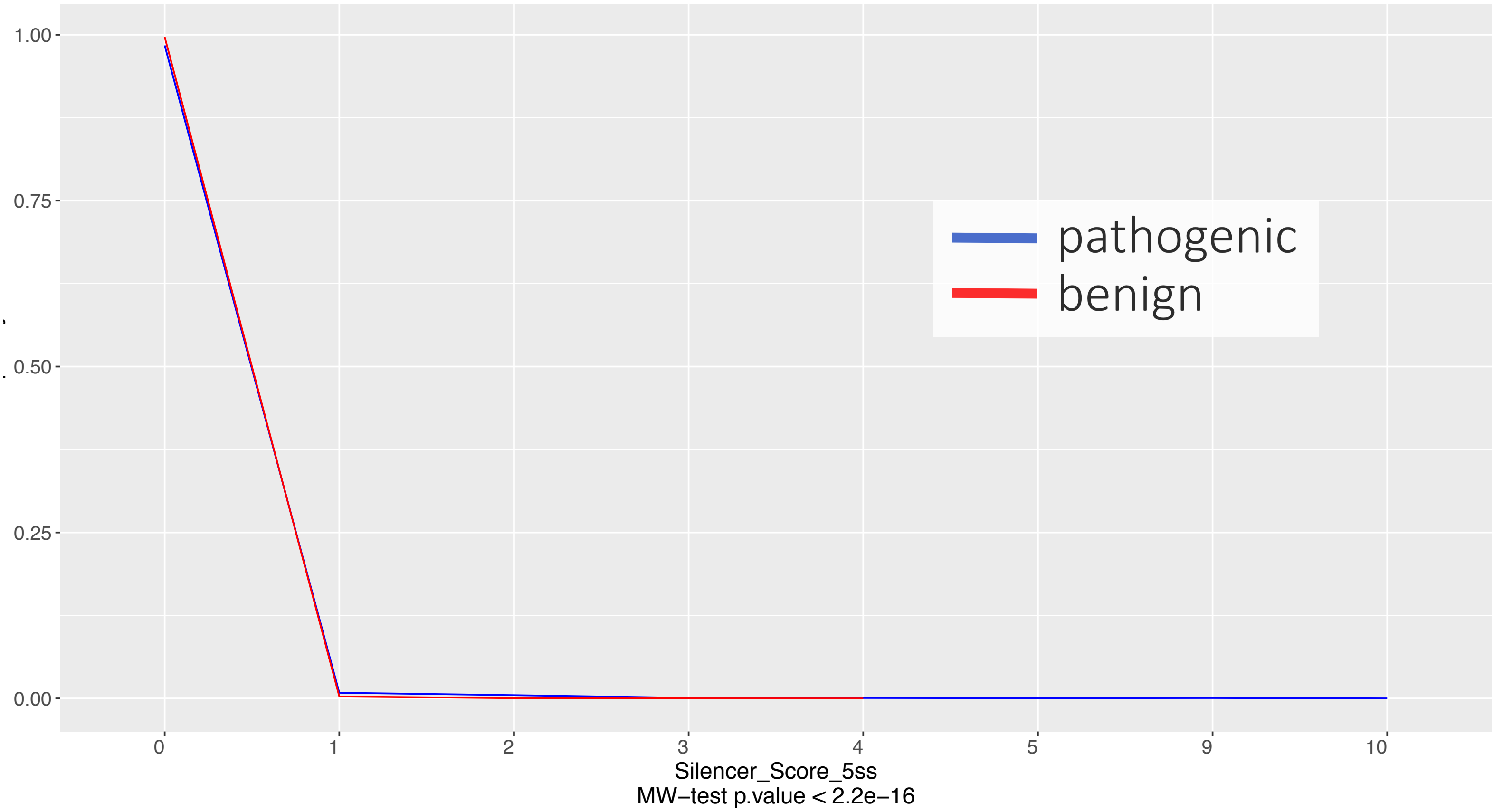

Supplementary Figure 14. Frequency distribution of the 5’ss Silencer Score (feature F10) for ExAC 1.46M synonymous variants. Frequencies are presented for TraP-predicted pathogenic variants (blue, TraP ≥ 0.459) and TraP-predicted benign variants (red, TraP < 0.459). The X-axis represents the 5’ss Silencer Score. The score is a sum of difference across all transcripts between the reference computed 5’ splice site PSSM score and the alternative 5’ splice site PSSM score in case the following conditions apply: 1) the variant is within the splice site region and 2) the new splice site is stronger than the original splice site. Corresponding Mann Whitney U test is used to compare benign and pathogenic variant distributions (bottom line).

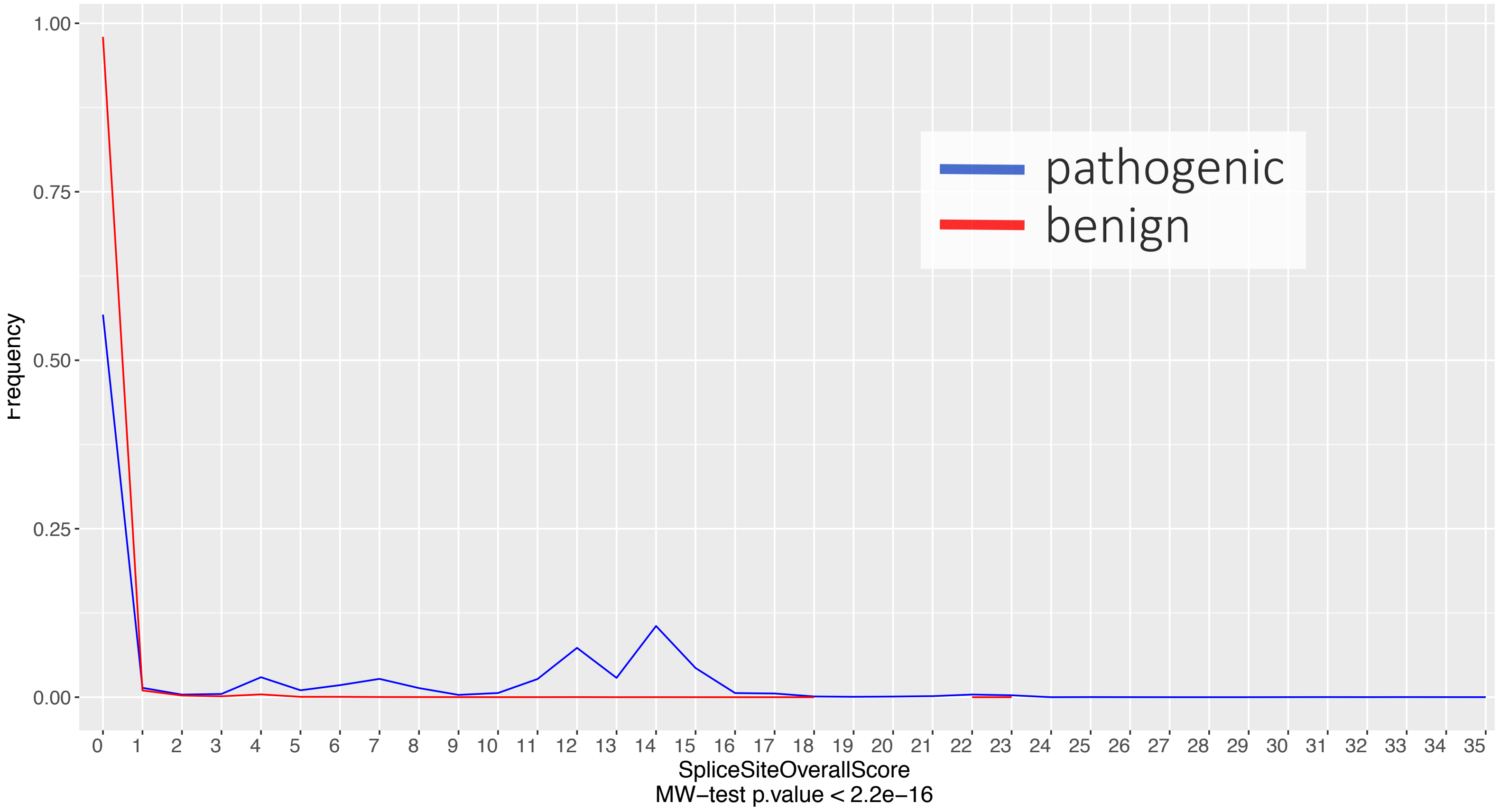

Supplementary Figure 15. Frequency distribution of the Splice Site Overall Score (feature F11) for ExAC 1.46M synonymous variants. Frequencies are presented for TraP-predicted pathogenic variants (blue, TraP ≥ 0.459) and TraP-predicted benign variants (red, TraP < 0.459). The X-axis represents the Splice Site Overall score: the sum of the variant effect on both 3' and 5' splice sites (F9 and F10) across all transcripts of the harboring gene. Corresponding Mann Whitney U test is used to compare benign and pathogenic variant distributions (bottom line).

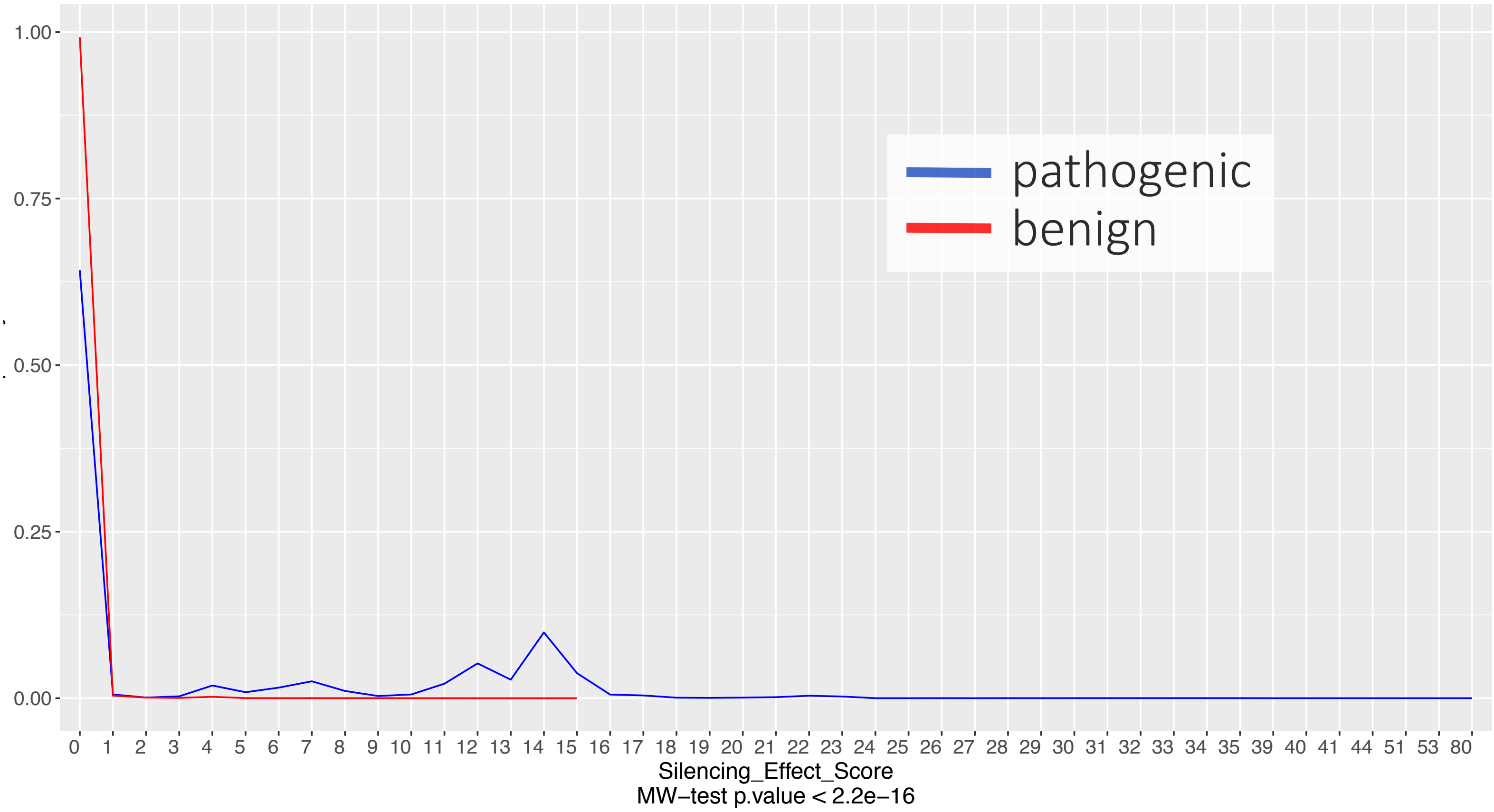

Supplementary Figure 16. Frequency distribution of the Silencing Effect Score (feature F12) for ExAC 1.46M synonymous variants. Frequencies are presented for TraP-predicted pathogenic variants (blue, TraP ≥ 0.459) and TraP-predicted benign variants (red, TraP < 0.459). The X-axis represents the Silencing Effect Score: the overall difference between reference splice site PSSM score and new splice site score combining the information from both 3' and 5' splice sites across all transcripts. Corresponding Mann Whitney U test is used to compare benign and pathogenic variant distributions (bottom line).

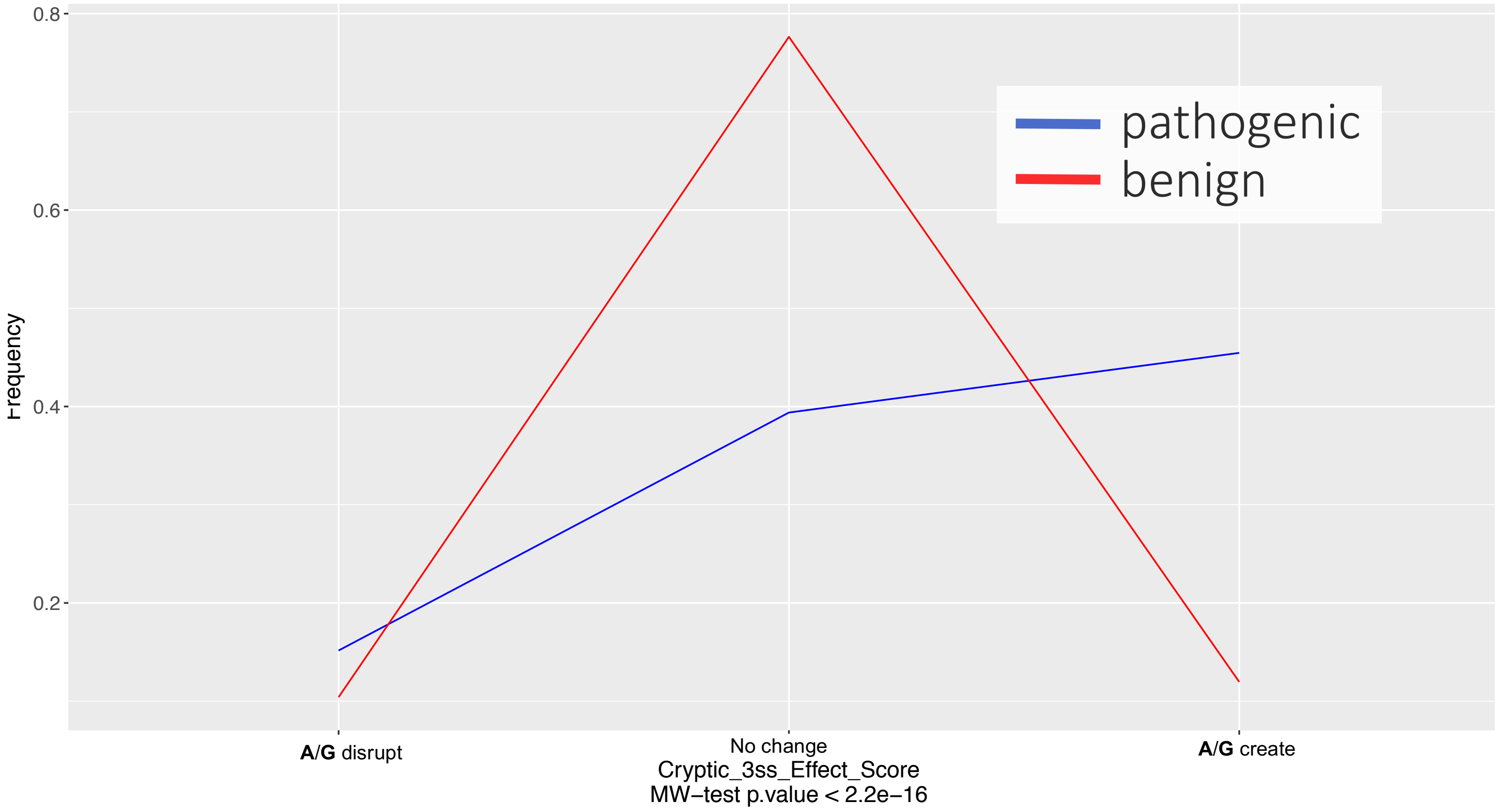

Supplementary Figure 17. Frequency distribution of the Cryptic 3'ss Effect Score (feature F13) for ExAC 1.46M synonymous variants. Frequencies are presented for TraP-predicted pathogenic variants (blue, TraP ≥ 0.459) and TraP-predicted benign variants (red, TraP < 0.459). The X-axis represents the Cryptic 3'ss Effect Score: a score representing the PSSM score difference between a newly created/disrupted 3' splice site and the original sequence at that position. Corresponding Mann Whitney U test is used to compare benign and pathogenic variant distributions (bottom line).

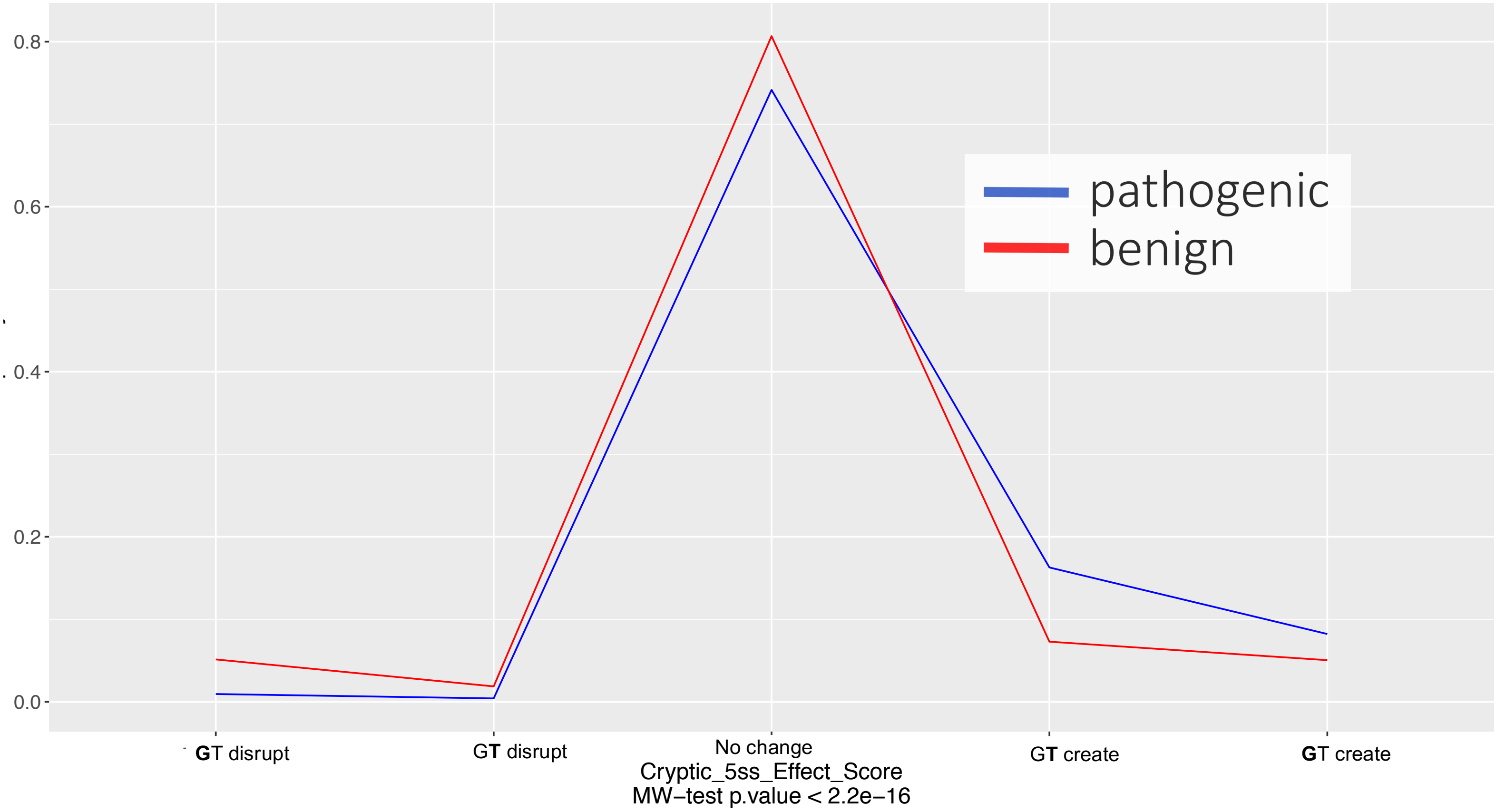

Supplementary Figure 18. Frequency distribution of the Cryptic 5'ss Effect Score (feature F14) for ExAC 1.46M synonymous variants. Frequencies are presented for TraP-predicted pathogenic variants (blue, TraP ≥ 0.459) and TraP-predicted benign variants (red, TraP < 0.459). The X-axis represents the Cryptic 5'ss Effect Score: a score representing the PSSM score difference between a newly created/disrupted 5' splice site and the original sequence at that position. Corresponding Mann Whitney U test is used to compare benign and pathogenic variant distributions (bottom line).

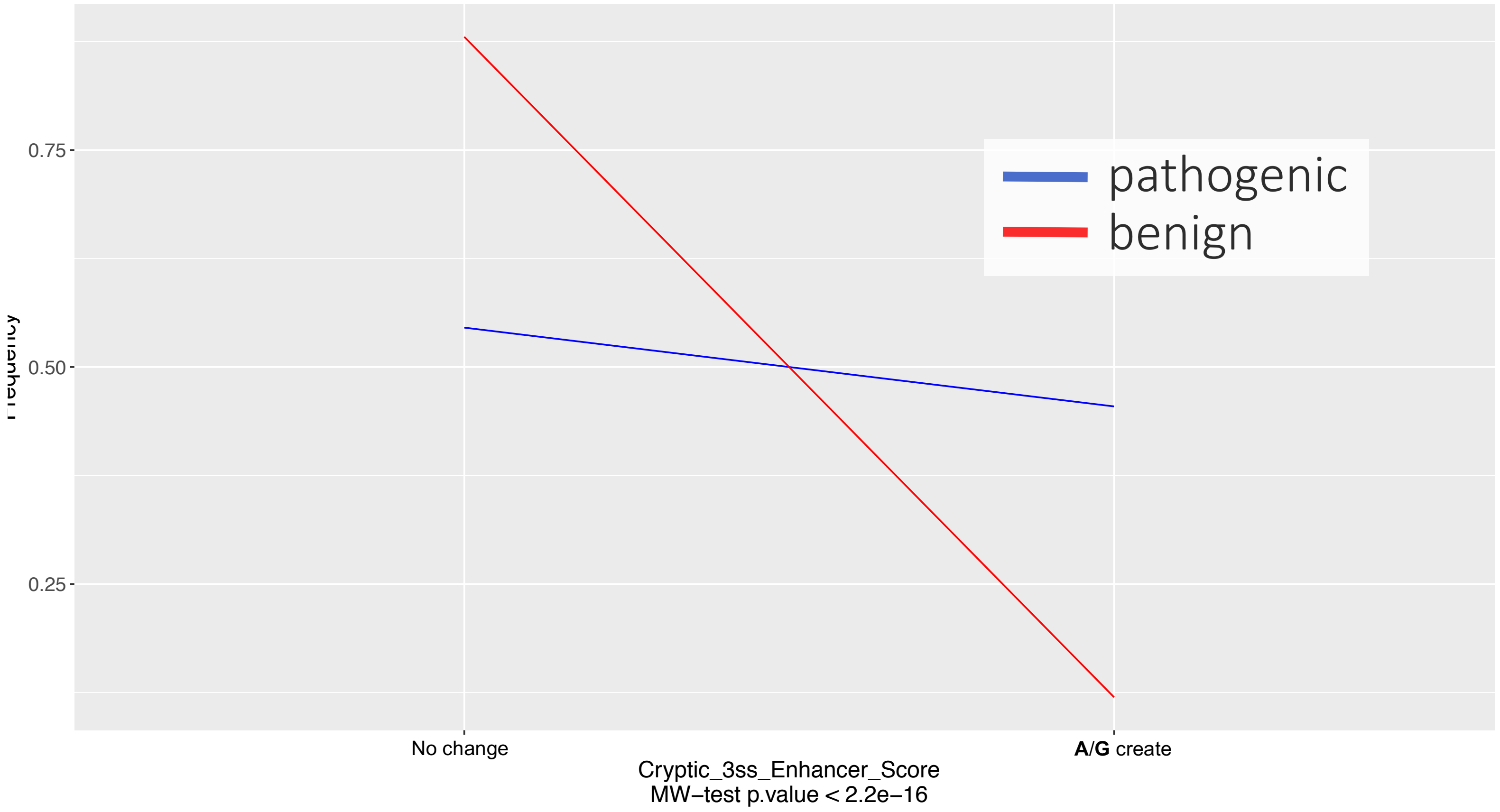

Supplementary Figure 19. Frequency distribution of the Cryptic 3'ss Enhancer Score (feature F15) for ExAC 1.46M synonymous variants. Frequencies are presented for TraP-predicted pathogenic variants (blue, TraP ≥ 0.459) and TraP-predicted benign variants (red, TraP < 0.459). The X-axis represents the Cryptic 3'ss Enhancer Score: this is similar to features F13, but with only positive differences examined, thus setting a zero value to features of variants that create cryptic splice sites that are weaker than the original sequence at that position. Corresponding Mann Whitney U test is used to compare benign and pathogenic variant distributions (bottom line).

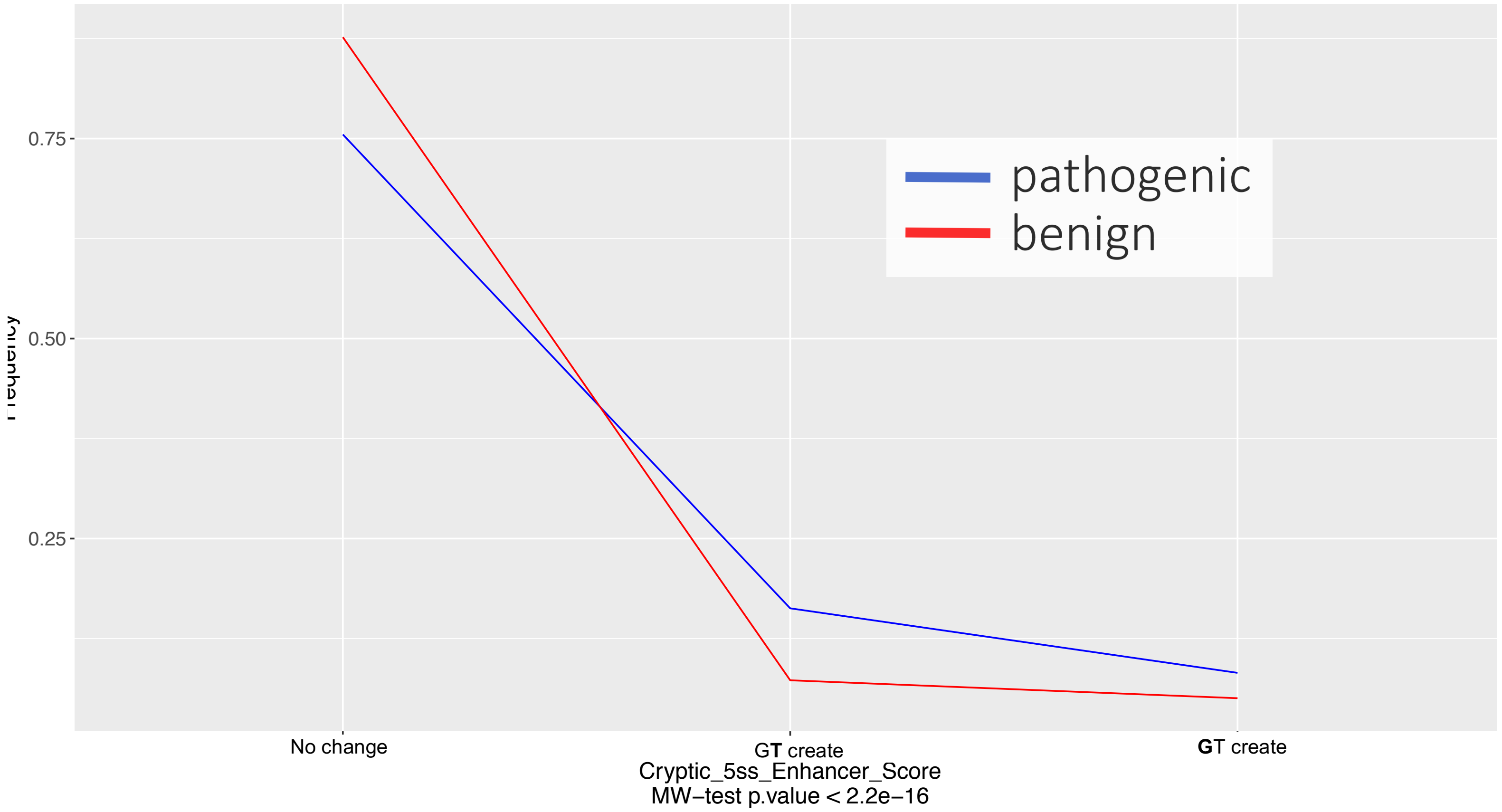

Supplementary Figure 20. Frequency distribution of the Cryptic 5'ss Enhancer Score (feature F16) for ExAC 1.46M synonymous variants. Frequencies are presented for TraP-predicted pathogenic variants (blue, TraP ≥ 0.459) and TraP-predicted benign variants (red, TraP < 0.459). The X-axis represents the Cryptic 5'ss Enhancer Score: this is similar to feature F14, but with only positive differences examined, thus setting a zero value to features of variants that create cryptic splice sites that are weaker than the original sequence at that position. Corresponding Mann Whitney U test is used to compare benign and pathogenic variant distributions (bottom line).

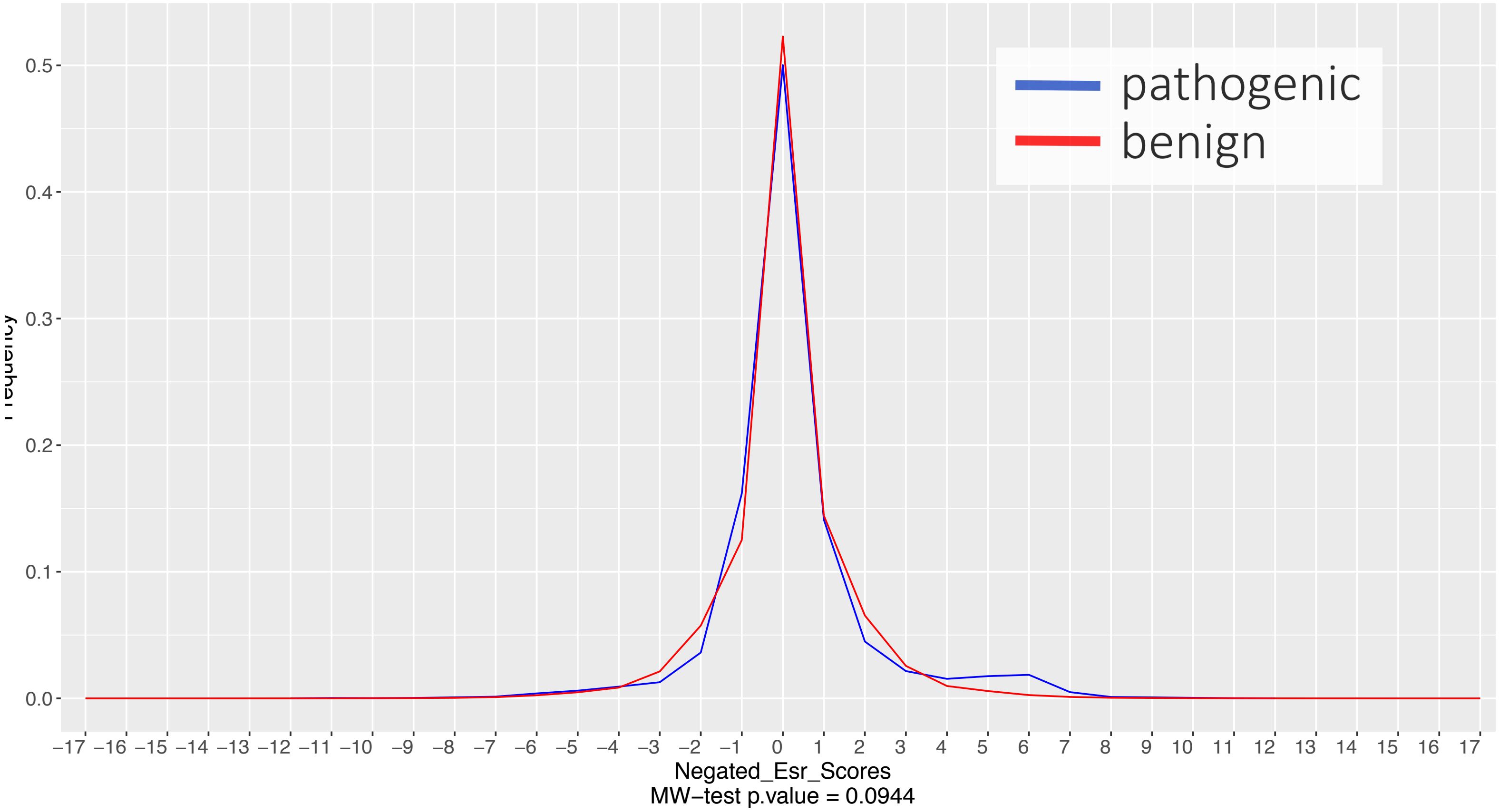

Supplementary Figure 21. Frequency distribution of the Negated ESR Score (feature F17) for ExAC 1.46M synonymous variants. Frequencies are presented for TraP-predicted pathogenic variants (blue, TraP ≥ 0.459) and TraP-predicted benign variants (red, TraP < 0.459). The X-axis represents the Negated ESR Score: the overall tendency of the variant to either silencing or enhancing, calculated as subtraction of the enhancing events caused by the variant from the silencing events caused by it. Corresponding Mann Whitney U test is used to compare benign and pathogenic variant distributions (bottom line).

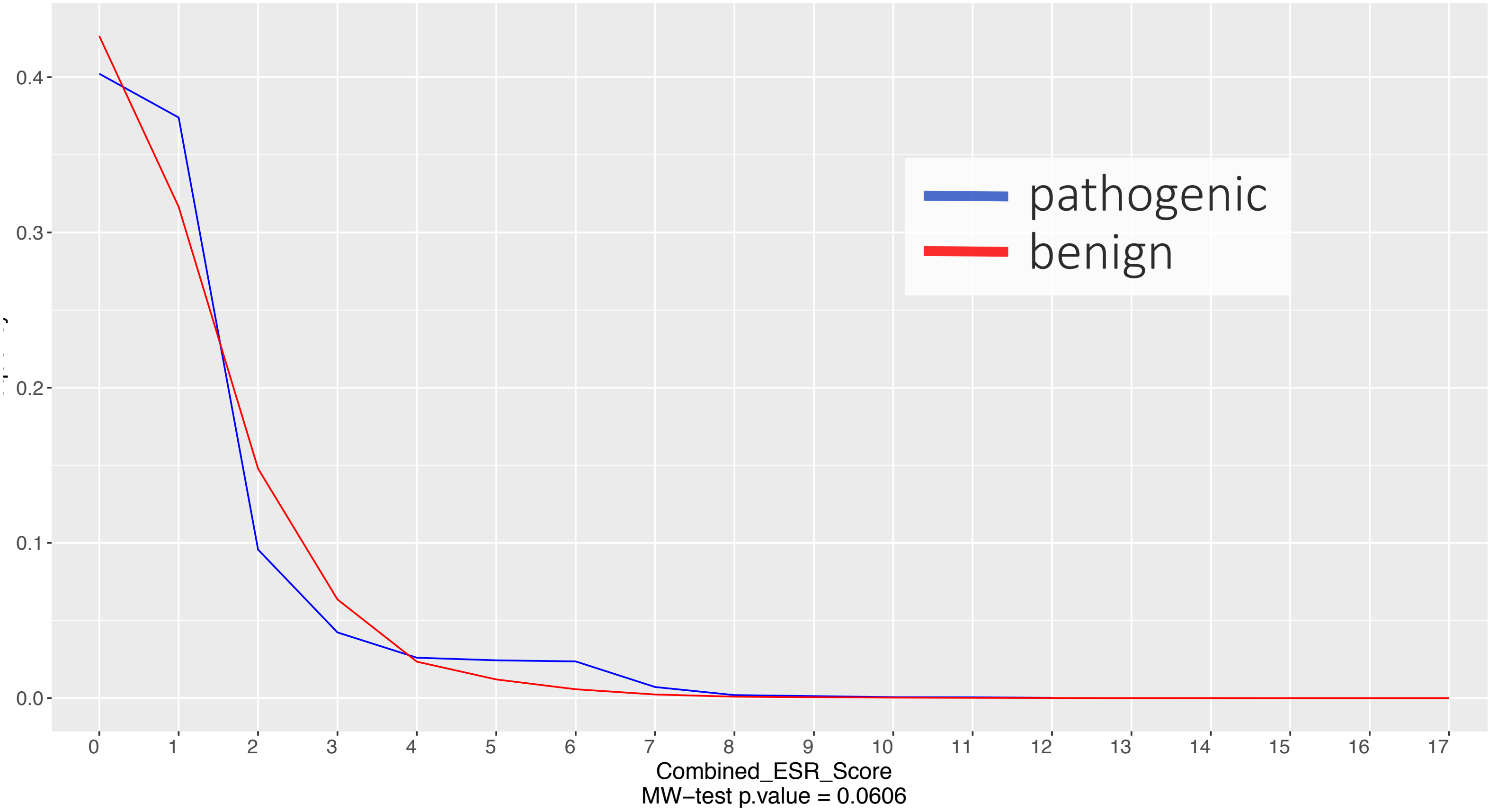

Supplementary Figure 22. Frequency distribution of the Combined ESR Score (feature F18) for ExAC 1.46M synonymous variants. Frequencies are presented for TraP-predicted pathogenic variants (blue, TraP ≥ 0.459) and TraP-predicted benign variants (red, TraP < 0.459). The X-axis represents the Combined ESR Score: the overall disturbance of regulatory sequences caused by the variant, calculated as the addition of enhancing and silencing ESR events caused by the variant. Corresponding Mann Whitney U test is used to compare benign and pathogenic variant distributions (bottom line).

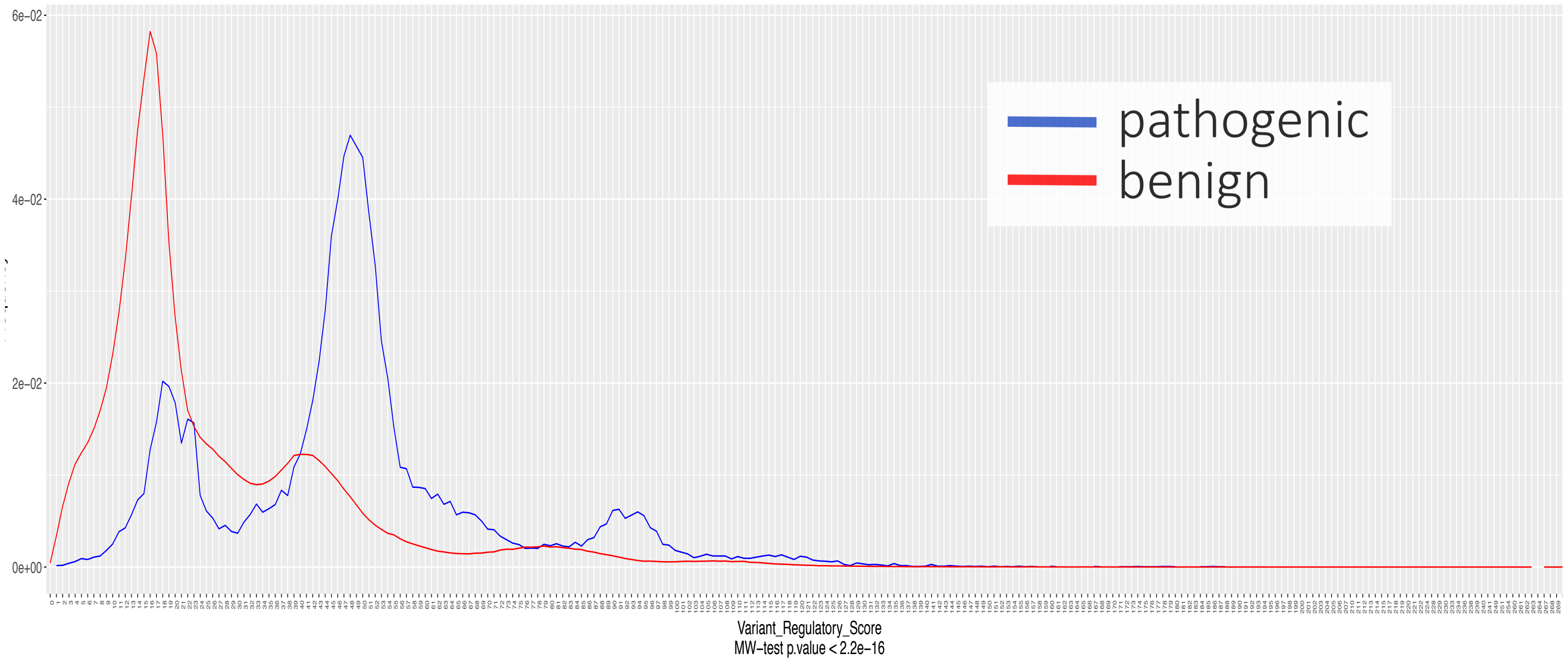

Supplementary Figure 23. Frequency distribution of the Variant Regulatory Score (feature F19) for ExAC 1.46M synonymous variants. Frequencies are presented for TraP-predicted pathogenic variants (blue, TraP ≥ 0.459) and TraP-predicted benign variants (red, TraP < 0.459). The X-axis represents the Variant Regulatory Score: a score that combines all the effects caused by the variant that do not directly change the splice site region. Corresponding Mann Whitney U test is used to compare benign and pathogenic variant distributions (bottom line).

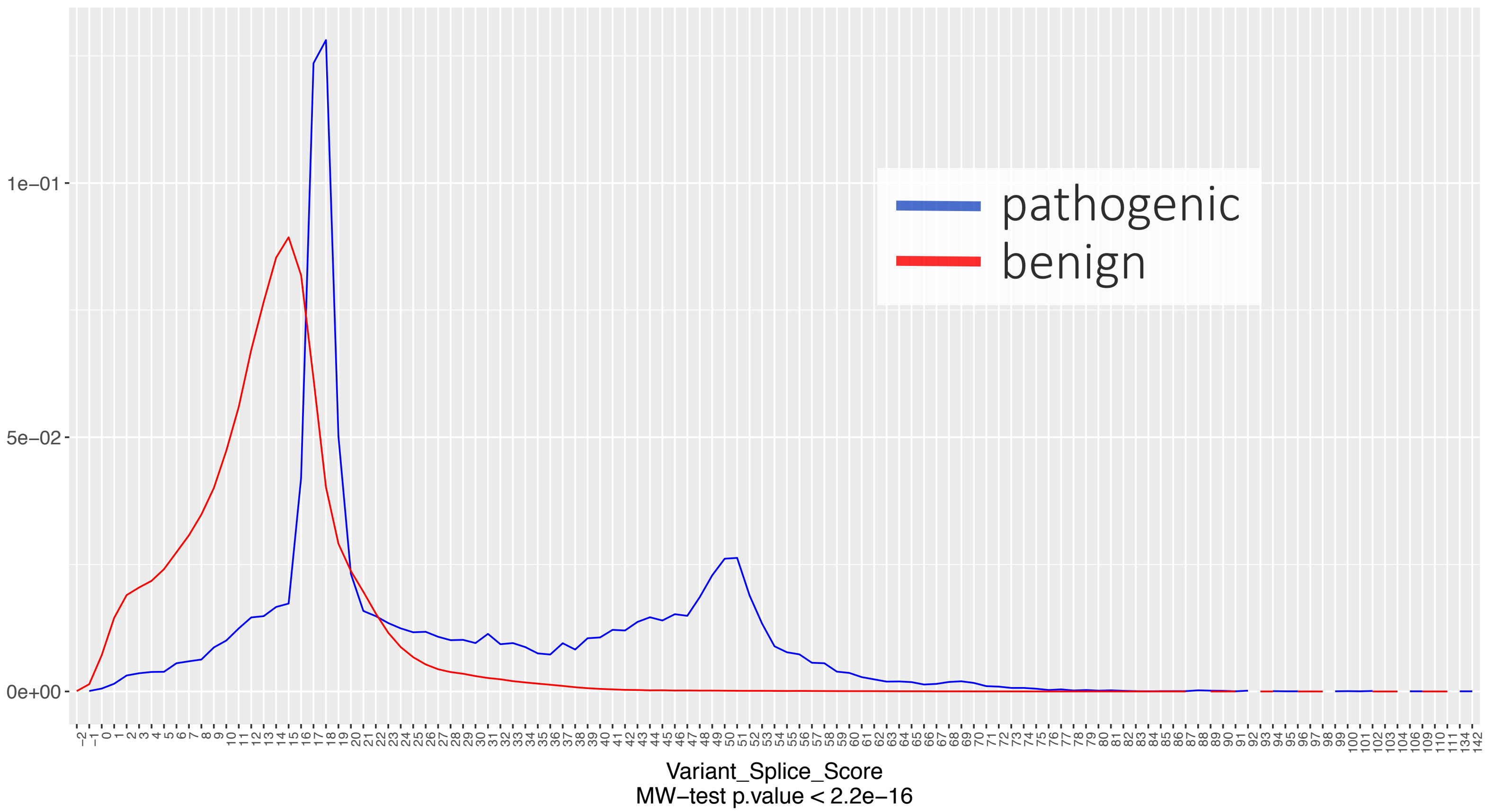

Supplementary Figure 24. Frequency distribution of the Variant Splice Score (feature F20) for ExAC 1.46M synonymous variants. Frequencies are presented for TraP-predicted pathogenic variants (blue, TraP ≥ 0.459) and TraP-predicted benign variants (red, TraP < 0.459). The X-axis represents the Variant Splice Score: the combined effects and interactions of regulatory and splice region features. Corresponding Mann Whitney U test is used to compare benign and pathogenic variant distributions (bottom line).

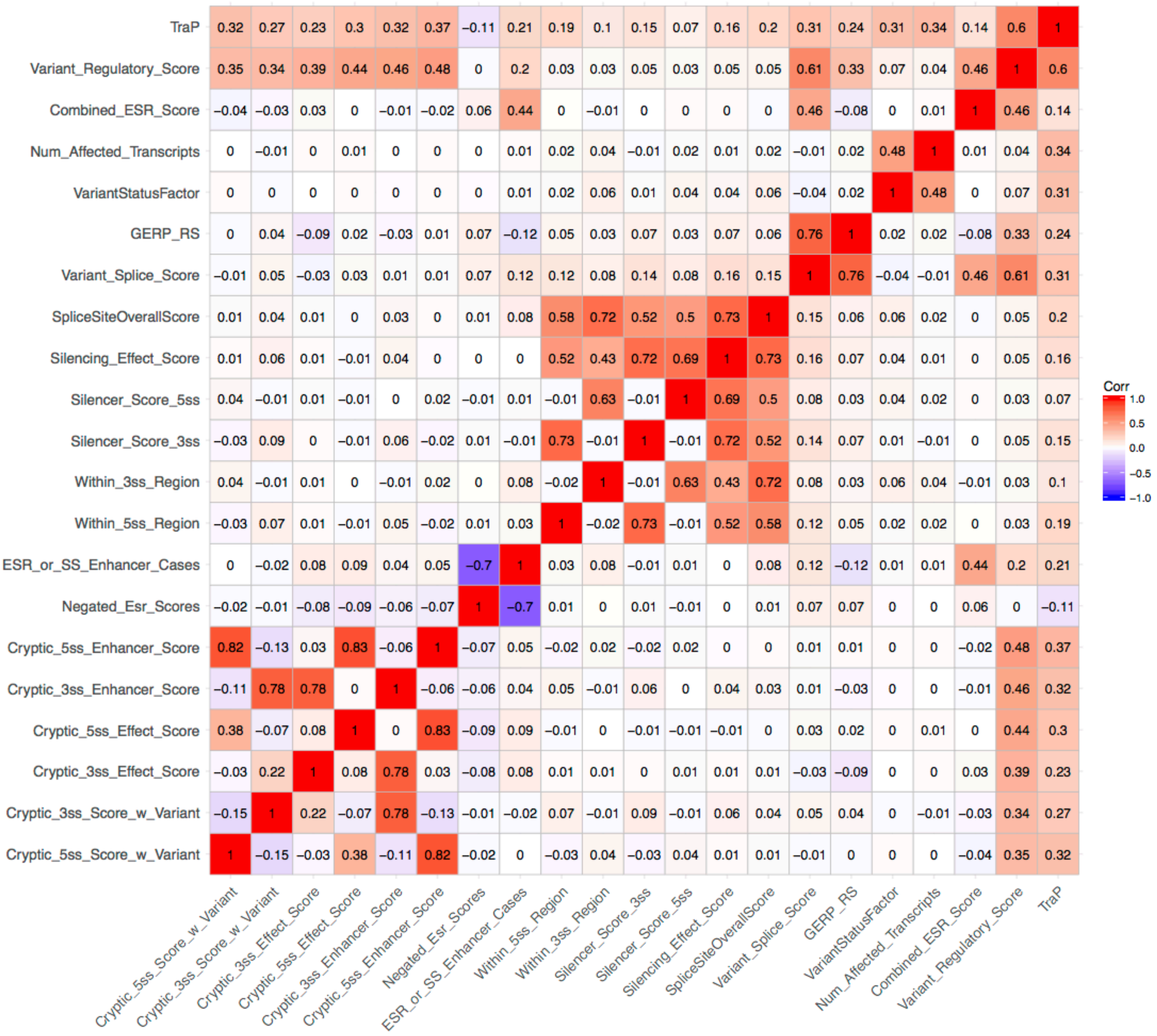

Supplementary Figure 25. Correlation matrix for TraP features. A Spearman correlation matrix incorporating all 20 features used for the final TraP model and calculated for the ExAC 1.46M synonymous variants.

Supplementary References

1. Xiong, H.Y. *et al.* RNA splicing. The human splicing code reveals new insights into the genetic determinants of disease. *Science* **347**, 1254806 (2015).
2. Buske, O.J., Manickaraj, A., Mital, S., Ray, P.N. & Brudno, M. Identification of deleterious synonymous variants in human genomes. *Bioinformatics* **29**, 1843-50 (2013).
3. Cartegni, L., Wang, J., Zhu, Z., Zhang, M.Q. & Krainer, A.R. ESEfinder: A web resource to identify exonic splicing enhancers. *Nucleic Acids Res* **31**, 3568-71 (2003).
4. Liu, H.X., Chew, S.L., Cartegni, L., Zhang, M.Q. & Krainer, A.R. Exonic splicing enhancer motif recognized by human SC35 under splicing conditions. *Mol Cell Biol* **20**, 1063-71 (2000).
5. Zhang, X.H. & Chasin, L.A. Computational definition of sequence motifs governing constitutive exon splicing. *Genes Dev* **18**, 1241-50 (2004).
6. Schwartz, S., Hall, E. & Ast, G. SROOGLE: webserver for integrative, user-friendly visualization of splicing signals. *Nucleic Acids Res* **37**, W189-92 (2009).
